# Transcriptome and Metabonomics Combined Analysis Revealed the Defense Mechanism Involved in Hydrogen-Rich Water-Regulated Cold Stress Response of *Tetrastigma hemsleyanum*

**DOI:** 10.3389/fpls.2022.889726

**Published:** 2022-06-23

**Authors:** Yuxiu Liu, Junjie Pan, Sui Ni, Bincong Xing, Kejun Cheng, Xin Peng

**Affiliations:** ^1^Ningbo Municipal Hospital of TCM, Affiliated Hospital of Zhejiang Chinese Medical University, Ningbo, China; ^2^School of Marine Sciences, Ningbo University, Ningbo, China; ^3^Chemical Biology Center, Lishui Institute of Agriculture and Forestry Sciences, Lishui, China; ^4^Zhejiang Provincial Key Laboratory of Resources Protection and Innovation of Traditional Chinese Medicine, Zhejiang A&F University, Hangzhou, China

**Keywords:** *Tetrastigma hemsleyanum*, cold stress, hydrogen-rich water, physiological and biochemical, RNA-seq, metabolite profiling

## Abstract

The poor resistance to cold stress conditions has become the bottleneck problem in *Tetrastigma hemsleyanum* (*T. hemsleyanum*) planting industry. Exogenous hydrogen (H_2_) plays an important role in improving stress resistance in plants. However, the key factors and regulatory network of plants in response to hydrogen-rich water (HRW) treatment under environmental stress are not clear. Here, we conducted integrative analyses of metabolome and transcriptome profiles to reveal the defense mechanism involved in the HRW-regulated cold stress response of *T. hemsleyanum*. The application of 75% HRW could alleviate stress damage by decreasing stomatal apparatus density and significantly increasing photosynthetic efficiency and mitigating physiological indexes of resistance, such as Pn, Cond, MDA, SOD, etc., which were changed by cold stress conditions. A total of 7,883 DEGs and 439 DEMs were identified. DEGs were the most relevant to phenylpropanoid, isoflavonoid, monoterpenoid, and flavonoid biosynthesis pathways. Using gene co-expression analysis (WGCNA), we identified one gene module that showed a strong correlation between total antioxidant capacity and transpiration rate. Trend analysis indicated that the phenylpropanoid biosynthesis pathway played a major role in the transcription and metabolism process of HRW treatment under cold stress. Based on the integrated analysis of genes and metabolites, the results showed cold stress upregulated the expression of PAL, CHS, COMT, CCR, AtBG1, etc., resulting in the accumulation of coniferyl alcohol and eriodictyol contents in *T. hemsleyanum* under cold stress, but the 75% HRW treatment could attenuate the enhancement. The study not only identified the main strategy of HRW protection against cold stress but also provided candidate genes for flavonoid biosynthesis, so as to better improve cold tolerance through molecular breeding techniques.

## Introduction

*Tetrastigma hemsleyanum* Diels et Gilg (*T. hemsleyanum*) belongs to the Vitaceae family, which is mainly distributed in the southwestern regions of China. It is included in the new group of “eight famous herbal drugs in Zhejiang” genuine medicinal materials. The previous studies indicated that *T. hemsleyanum* contained flavonoids, terpenoids, amino acids, and volatile oil (Zhu et al., 2020). Among them, flavonoids are important physiological active substances and are directly related to the antiviral, hepatoprotective, anti-inflammatory, and immune regulation potential of *T. hemsleyanum* (Ji et al., 2020). Recently, *T. hemsleyanum* plants are on the verge of extinction due to overexploitation (Peng et al., 2015). Therefore, it is imperative to strengthen the domestication and artificial cultivation of *T. hemsleyanum* to reduce the dependence on wild resources. However, the artificial cultivation of *T. hemsleyanum* is markedly restricted by poor cold resistance. Cold damage has become a major environmental factor affecting the growth and geographical distribution of *T. hemsleyanum*. On the other hand, osmotic stress, free radical burst, and material metabolism disorder might occur in the cells under low temperatures, which would seriously affect the quality of medicinal materials. In addition to cultivating new cold-resistant varieties, another simple and effective method to enhance the cold resistance of *T. hemsleyanum* is by using exogenous substances.

Hydrogen has long been considered as a physiological inert gas. In 2007, Nature Medicine reported for the first time that H_2_ alleviated oxidative damage caused by cerebral artery ischemia-reperfusion in rats by selectively scavenging hydroxyl radicals (Ohsawa et al., 2007), which aroused the extensive interest of researchers. At present, research on the botanical effects of H_2_, which started late, has also made some progress. Many researchers believe that hydrogen is likely to be another important bioactive signal transduction gas molecule after nitric oxide (NO), carbon monoxide (CO), and hydrogen sulfide (H_2_S) (Jin et al., 2013; Zeng et al., 2014; Li et al., 2018; Hancock and Russell, 2021). Because hydrogen is colorless, odorless, and has potential advantages of strong permeability and quick diffusibility, some people speculate that it will not cause environmental pollution after treatment.

Studies have shown that abiotic stress can lead to the release of endogenous H_2_ in plants, and exogenous hydrogen-rich water (HRW) treatment can also stimulate the production of endogenous H_2_ (Cao et al., 2017), which can mitigate the adverse effects of abiotic stress. Research by Xie and Dai et al. showed that HRW treatment enhanced plant resistance to salt (Xie et al., 2012) and heavy metals (Dai et al., 2017). Zhao et al. (2017) revealed that exogenous HRW (25–75% concentration) treatment improved aluminum tolerance in maize seedlings, with the most significant effect being observed for 75% HRW. The synthesis of reactive oxygen species was reversed to a great extent under drought conditions by 75% HRW treatment, and the drought tolerance of *Arabidopsis thaliana* was improved to a great extent (Xie et al., 2014). Our previous experiment also confirmed that 75% HRW was the most suitable concentration for improving the cold resistance of *T. hemsleyanum*.

However, the research on HRW is still in its infancy, with studies mainly focusing on the exploration of its biological effects. The stress-relieving effects of H_2_ are only carried out on a few model plants and crops. Most of the indexes are related to primary metabolisms, such as growth, development, and organ differentiation. Moreover, secondary metabolites in plants are widely found to be closely related to their stress resistance and act as key factors in determining the quality of medicinal plants. However, studies regarding the effect of exogenous H_2_ on the secondary metabolism of plants are rare. Herein, we analyzed the effects of exogenous H_2_ on the freezing damage and quality changes in *T. hemsleyanum* under cold stress based on the analysis of physiological and biochemical indexes and explored the possible molecular mechanisms of HRW involved in alleviating cold damage by comparative transcriptomic and metabolomic approaches. It would provide a theoretical basis for enriching the theoretical research on the metabolism and function of plant gas signal molecules and for expanding the application of HRW in the artificial planting of medicinal plants.

## Materials and Methods

### Preparation of 75% HRW

Hydrogen-rich water was prepared using a high-purity hydrogen generator (SHC-300, Shandong Sykes Hydrogen Energy Co., Ltd.) to prepare 99.99% (v/v) pure hydrogen electrolytic water, which was passed into 1 L of deionized water (pH 6.0, 25°C) at a rate of 300 ml/min for 30 min until saturation (Wang et al., 2019), followed by rapid dilution of the saturated solution of hydrogen to the desired concentration (75%, v/v). The hydrogen concentration in the freshly prepared HRW was measured to be about 0.22 ppm using a dissolved hydrogen tester (ENH-2000, Trustlex, Japan), which could be maintained at least for about 12 h at 25°C (Jin et al., 2016).

#### Plant Materials and Cold Stress Treatments

The cutting seedlings of *T. hemsleyanum* were harvested in planting bases of Ningbo Shengwang Co., Ltd. (Ningbo, China), and the leaves with uniform growth, regular shape, and healthy leaves were selected and divided into three groups, including the TH-0% group (ultrapure water was sprayed, 0°C), TH-75% group (the same volume of 75% HRW was sprayed, 0°C), and CK group (the same volume of ultrapure water was sprayed, 25°C). About 10 ml of ultrapure water or HRW was sprayed on each seedling once every 0.5 h. The seedlings were taken from the same plant at 0, 4, 8, and 12 h, respectively, and the physiological responses were evaluated. Then, based on the results of photosynthetic and physiological parameters, the stress treatment periods were selected. The leaves were sampled and cut into 1 cm^2^ rectangles after 8 h of treatment, which were further used for transcriptome and metabolome analysis. Three independent biological replicates were performed for each treatment, and each replicate contained 10 mature leaves (the second fully expanded leaves from the top). All samples were immediately frozen in liquid nitrogen and stored at −80°C.

#### Scanning Electron Microscope Sample Preparation and Observation

Leaves of TH-0%, TH-75%, and CK groups were cut into pieces of about 3 mm × 3 mm size with a blade, quickly placed into 2.5% glutaraldehyde solution, fixed for 2 h at room temperature after pumping air to make the tissue sink, rinsed using the phosphoric acid buffer for three times, subjected to gradient dehydration with ethanol (50, 70, 80, 90, 95, and 100%), dehydrated with anhydrous ethanol, and dried using critical point dryer (CPD300, Leica Microsystems, Wetzlar, Germany). Then, the sample was pasted on the cover glass, put into the vacuum spray machine for spraying, and finally observed and photographed by scanning electron microscope (HITACHI SU3900, Shimadzu Corporation, Tokyo, Japan). The number of stomata was observed under the scanning electron microscope, and three replicate samples were used for each treatment group. The stomatal density (pieces/mm^2^ = number of stomata in the field of view/field area) was calculated by measuring the field of view using a micrometer scale and calculating the field of view.

#### Determination of Photosynthetic and Physiological Parameters

Net photosynthetic rate (Pn), transpiration rate (Tr), stomatal conductance (Cond), and mesophyll intercellular CO_2_ (Ci) content were measured using LI-6400 portable photosynthesis meter (LI-6400XT, LI-Cor, Huntington Beach, CA, United States). The content of malondialdehyde (MDA) in the leaves was measured by the 2-thiobarbituric acid (TBA) method (Kong et al., 2016). Proline (PRO) was extracted with sulfosalicylic acid and reacted with acid ninhydrin to form a stable red product. Then the absorbance was measured at 520 nm. The content of PRO was calculated by constructing a standard curve (Dash et al., 2017). The superoxide dismutase activity (SOD) in the leaves was measured by the photochemical reduction of azoblue tetrazole (Giannopolitis and Ries, 1977). The content of soluble sugar (SS) was determined by the anthrone colorimetric method (Laurentin and Edwards, 2003). The total antioxidant capacity (T-AOC) was evaluated using the Total Antioxidant Capacity ELISA Kit (G0142F, Suzhou Grace Biotechnology Ltd., Suzhou, China).

#### RNA-Seq Data Analysis

RNA extraction, quantification, and transcriptome sequencing were carried out according to our previous method (Peng et al., 2019, 2021). We used NEBNext^®^ Ultra™ RNA Library Prep Kit for Illumina^®^ (NEB, United States) to generate sequencing libraries. The index coded samples were clustered on the cBot Cluster Generation System using TruSeq PE Cluster Kit v3-cBot-HS (Illumia). The library preparations were sequenced on an Illumina Hiseq platform, and paired-end reads were generated. Trinity software was used to perform transcriptome *de novo* assembly. We used DIAMOND-BLASTX v0.8.24 (Chialva et al., 2020) to compare the assembled transcript sequences using KEGG, GO, Swiss-Prot, KOG, and NR databases (Buchfink et al., 2015). After predicting the amino acid sequence of the transcript, HMMER software was used to compare with the Pfam database to obtain the annotation information of the transcript. The RNA-seq data were sequentially processed, and then the gene expression quantity was calculated by Bowtie2 to obtain the expression matrix (Langmead and Salzberg, 2012). EdgeR was used to detect the differential expression between the two groups. The log_2_ | FoldChange | > 2 and *P* values < 0.05 were identified as screening threshold for significant differences in expression.

#### Metabolite Extraction, Profiling, and Qualitative and Quantitative Analysis

Biological samples were freeze-dried in a vacuum freeze-dryer (ScientZ-100F Ningbo Scientz Biotechnology Ltd., Ningbo, China). A mixer with zirconia beads (MM 400, Retsch Technology, Haan, Germany) was used to crush and dry the samples for 1.5 min at 30 Hz. About 100 mg of freeze-dried powder was dissolved in 1.2 ml of 70% methanol solution, rotated every 30 min for 30 s, six times in total, and the sample was placed in a refrigerator at 4°**C** overnight. After centrifugation at 12,000 rpm for 10 min, the extract was filtered before (SCAA-104, 0.22 μm aperture; Shanghai Anpel, China^1^) subjecting to UPLC-MS/MS analysis. Metabolites were identified by m/Z values, retention time, and fragmentation patterns of standard products in the MetWare Biotechnology Co., Ltd. (Wuhan, China) database. Significantly regulated metabolites between the groups were determined by VIP ≥ 1 and absolute Log_2_FC (folding change) ≥ 1. VIP values were extracted from the OPLS-DA results, which also contained a score graph and a permutation graph, generated using the R software package MetaboAnalystR.

#### Co-expression Network Analysis With WGCNA

Co-expression networks were constructed by the WGCNA package in R from all the DEGs. (Langfelder and Horvath, 2008). WGCNA is a bioinformatic method. R software WGCNA package was used to establish the undirected correlation network, and soft threshold β = 1 was adopted for the screening purpose. The minimum number of module genes was 30, and the threshold of module combination was 0.25. A clustering tree was constructed and modules were divided according to the correlation of gene expression, and the data were used for the identification of gene modules involved in flavonoid metabolism. These gene modules were correlated with Pn, Ci, Cond, Tr, MDA, PRO, SOD, SS, and T-AOC parameters, and the correlation between gene modules and physiological and biochemical indexes was calculated by using a related phenotypic trait matrix.

#### Trend Analysis of DEGs

EdgeR software (Robinson et al., 2010) was used to analyze the differences in gene expression with biological duplication in each comparison group. The screening conditions for DEGs were FDR ≤ 0.05 and | Log_2_ Fold change | ≥ 1. Trend analysis was conducted on the common differentially expressed genes in each comparison group, and *P* ≤ 0.05 was set as the expression pattern with a significant difference in the software.

### Quantitative Real-Time PCR Analysis

Total RNA was extracted from *T. hemsleyanum* leaves according to the instructions of the TransZol Plant kit. The A_260_ and A_280_ values of the extracted RNA samples were measured by using a NanoDrop Micro UV spectrophotometer, and the sample concentrations were calculated. The extracted total RNA was stored in a refrigerator at −80°C. The relevant gene amplification primers were designed by Beacon Designer 7 and synthesized by Shanghai Generay Biotech Co (Table 1). The RNA obtained from three biological replicates of each sample was reverse-transcribed into complementary deoxyribonucleic acid (cDNA) using the TransScript II Probe One-Step kit, and the reverse transcription product was used as the template with MDH (malic dehydrogenase) gene as the internal reference gene. The SYBR Green RT-PCR kit from QIAGEN was used for fluorescence quantification using the FTC-3000 qPCR system (Canada Funglyn Biotech Co., Ltd.). The PCR reaction procedure was as follows: incubation at 95°C for 2 min, followed by 35 cycles at 95°C for 5 s and 60°C for 30 s. Three technical replicates were performed for each sample. The collected data were used to calculate the relative expression of genes using the 2^–ΔΔ*Ct*^ method. Each gene was tested in three biological replicates, with three technical repeats. The data were exhibited as the mean ± SD of three independent experiments.

**TABLE 1:** Primer sequences used for qRT-PCR for detecting gene expression.

Gene annotation	Tm/°C	Primer F (5′−3′)	Primer R (5′−3′)
Malic dehydrogenase (MDH)	60	TGTTGCTACGACTGATGT	CCTGAGACTTGTAGATGGAA
Beta-glucosidase (AtBG1)	60	GCTATAACAGATGATGATCCAG	CATACACAGAAGCAGTTGAA
Phenylalanine ammonia-lyase (PAL)	60	GGTGAGACGCTTACGATA	GATTCTGTTCCATTCCCAAA
Cinnamoyl-CoA reductase (CCR)	60	TGTGATACCGAGAAGAAGAA	GGTCGTCTCTCCAAATGA
Caffeic acid 3*-O-*methyltransferase (COMT)	60	TGAAGAAGAGGCACAGAA	GGTGAGGAAGTAGAGAAGG
Chalcone synthase (CHS)	60	ACCCAAGAGATTAGCAAGT	CTAAGAGCGAGCACAAGA
Chalcone reductase (CHR)	60	CAGGTGATGGATTAGATTGG	GCGGATAGGGATTTGAGA

#### Statistical Analysis

The normalized expression of the gene was calculated as reads per kilobase per million reads (RPKM) value. The transcriptomic data are available in the NCBI Gene Expression Omnibus repository^2^ under accession number PRJNA816565. We used the R package to analyze the differentially expressed genes (DEGs), and the significance was judged based on the false discovery rate ≤ 0.01 and the absolute value of log_2_Ratio ≥ 1. WGCNA analysis was performed using the R package “WGCNA.” Hierarchical clustering analysis of metabolites was performed using the expander R package “Complex Heatmap.” The software program SPSS, version 26.0 (SPSS Inc., Chicago, IL, United States) was used for statistical analysis, and one-way analysis of variance (ANOVA) was performed to measure the difference between the mean values under Duncan’s multiple range test (DMRT) at a significant level of 0.05. The results are presented as the mean ± standard deviation values. Processed data were translated into graphs using GraphPad Prism, version 6.0.

## Results

### HRW Alleviated the Damage Degree of *T. hemsleyanum* Seedlings Under Cold Stress

**FIGURE 1:**
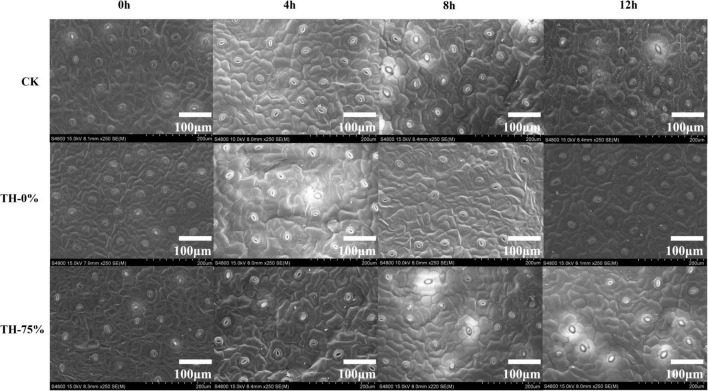
The scanning electron microscopy of leaf surfaces in CK, TH-0%, and TH-75% at 0, 4, 8, and 12 h.

The leaves of each treatment group were observed by scanning electron microscope. It was found that the density of the stomatal apparatus on the leaf surface changed in different degrees under different exposure times to low-temperature stress conditions (Figure 1).

In the TH-0% group, the stomatal density of *T. hemsleyanum* leaves hardly changed from 0 to 8 h, and then increased significantly at 12 h under cold stress, which showed that the cold stress exceeded the tolerance of plants, and hence the stomatal density increased significantly (Table 2). However, the stomatal apparatus density of TH-75% changed barely from 0 to 12 h, indicating that the application of HRW could alleviate stress damage induced by the external environment by decreasing stomatal apparatus density under cold stress.

**TABLE 2:** The stomatal density of leaf surfaces in CK, TH-0%, and TH-75% at 0, 4, 8, and 12 h.

Groups	Time/(h)	Stomata density/(pcs/mm^2^)
CK	0	172 ± 14a
	4	178 ± 9a
	8	169 ± 9a
	12	175 ± 5a
TH-0%	0	178 ± 24a
	4	175 ± 14a
	8	169 ± 24a
	12	199 ± 14b
TH-75%	0	175 ± 5a
	4	172 ± 10a
	8	178 ± 9a
	12	169 ± 24a

*Different letters between different time intervals for each treatment indicate significant differences (P < 0.05), and the same letters indicate no significant differences (P ≥ 0.05).*

**FIGURE 2:**
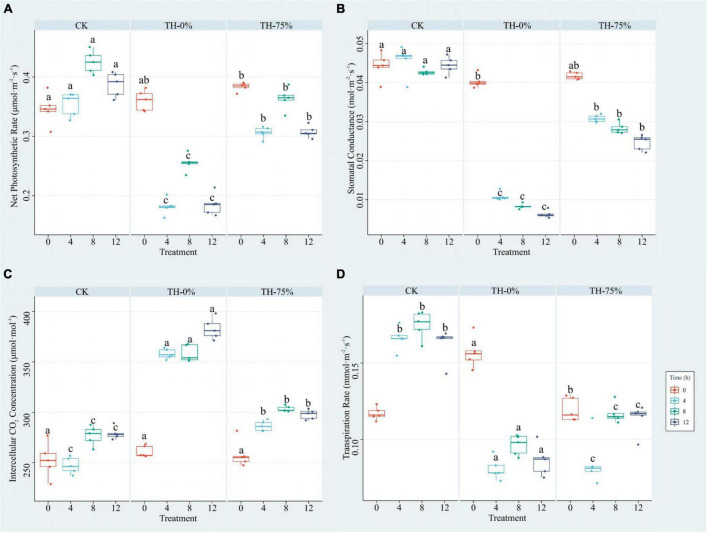
Pn **(A)**, Cond **(B)**, Ci **(C),** and Tr **(D)** of *T. hemsleyanum* in CK, TH-0%, and TH-75% groups at 0, 4, 8, and 12 h. Data are mean ± SE (*n* = 5). Data labeled with different lowercase letters are significantly different at *P* < 0.05 level.

The results showed that Pn, Cond, and Tr values of the TH-0% group were reduced by 39.9–52.2%, 76.2–85.8%, and 44.9–51.3%, respectively, compared to the CK group (Figures 2A,B,D), whereas Ci was significantly increased by 29.4–44.8% (Figure 2C). Pn, Cond, and Tr values of the TH-75% group were significantly higher than those observed in the TH-0% group by 42.2–68.4%, 184.0–291.1%, and 5.6–32.3%, respectively. Ci of the TH-75% group was 29.4–44.8% lower than that observed in the TH-0% group. So, HRW treatment narrowed the gap between the cold stress treatment and CK groups. These results indicated that HRW treatment significantly enhanced photosynthetic efficiency under cold stress (*P* < 0.05).

**FIGURE 3:**
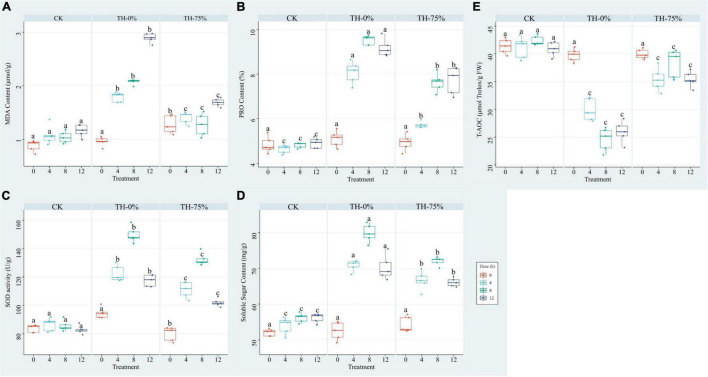
MDA **(A)**, PRO **(B)**, SOD **(C)**, SS **(D),** and T-AOC **(E)** of *T. hemsleyanum* in CK, TH-0%, and TH-75% groups at 0, 4, 8, and 12 h. Data are mean ± SE (*n* = 5). Data labeled with different lowercase letters are significantly different at *P* < 0.05 level.

The results showed that MDA, PRO, SOD, and SS values were increased in the TH-0% group by 66.0–148.8%, 74.2–98.1%, 41.7–75.1%, and 24.4–40.8%, respectively (Figures 3A–D), whereas T-AOC was significantly reduced by 27.3–42.0% (Figure 3E) when compared to the CK group. MDA, PRO, SOD, and SS values in the TH-75% group were significantly lower than those observed in the TH-0% group by 21.5–41.6%, 15.9–29.0%, 9.3–13.2%, and 4.7–8.7%, respectively. In contrast, the T-AOC of the TH-75% group was 18.4–55.8% higher than that of the TH-0% group. Overall, the content of MDA, PRO, SOD, and SS increased after cold stress treatment and declined after HRW treatment, while T-AOC showed a reverse tendency. After a comprehensive analysis, HRW treatment significantly narrowed the gap between CK and cold stress treatment groups.

### Analysis of Differentially Accumulated Secondary Metabolite in HRW-Treated *T. hemsleyanum* Under Cold Stress

**FIGURE 4:**
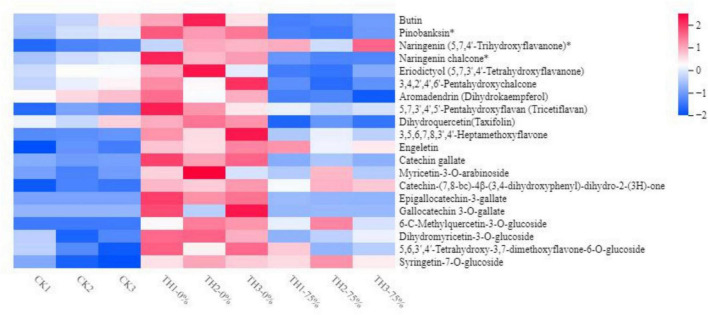
Heat map visualization of metabolites. The content of each flavonoid metabolite was normalized. Each sample is visualized in a single column, and each metabolite is represented by a single row. Red indicates high abundance, whereas low relative metabolites are shown in blue (the color key scale is provided on the right of the heatmap). The symbol * indicates that isomers of the metabolite, but specific isomers cannot be distinguished.

The widely targeted LC-MS/MS-based metabolome approach was adopted to analyze the metabolite changes between CK TH-0% groups and between TH-0% and TH-75% groups. The differentially accumulated metabolites (DAMs) in CK, TH-0%, and TH-75% groups were screened using | log_2_ fold change | ≥ 1 and *P*-value < 0.05. A total of 439 secondary metabolites were detected and divided into eight classes, including 177 flavonoids, 151 phenolic acids, 28 alkaloids, 23 lignans and coumarins, 20 tannins, and others. A total of 35 DAMs were identified in TH-0% vs TH-75% groups, which were divided into six categories, including 15 flavonoids, 8 phenolic acids, 7 terpenoids, 2 tannins, 2 other classes, and 1 alkaloid. A total of 15 DAMs were identified in CK vs TH-0% groups, which were divided into 5 categories, including 8 flavonoids, 3 phenolic acids, 2 alkaloids, 2 terpenoids, and 1 other class. It can be seen from Figure 4 that the content of flavonoid compounds increased significantly under cold stress compared to that observed in the CK group, while the increasing trend of flavonoids was significantly reduced by HRW treatment (*P* < 0.05).

The content of 6-C-methylquercetin-3*-O-*glucoside, syringetin-7*-O-*glucoside, and dihydromyricetin-3*-O-*glucoside in TH-0% was increased significantly in response to cold stress (*P* < 0.05), compared to CK. In particular, 6-C-methylquercetin-3*-O-*glucoside was almost undetectable in CK. The content of these DEMs in the TH-75% group was decreased by 74.0%, 66.8%, and 78.1%, respectively, when compared to the TH-0% group. In summary, we noticed that HRW treatment significantly reduced the increasing trend of flavonoid content induced by cold stress, particularly 6-C-methylquercetin-3*-O-*glucoside, syringetin-7*-O-*glucoside, and dihydromyricetin-3*-O-*glucoside.

**FIGURE 5:**
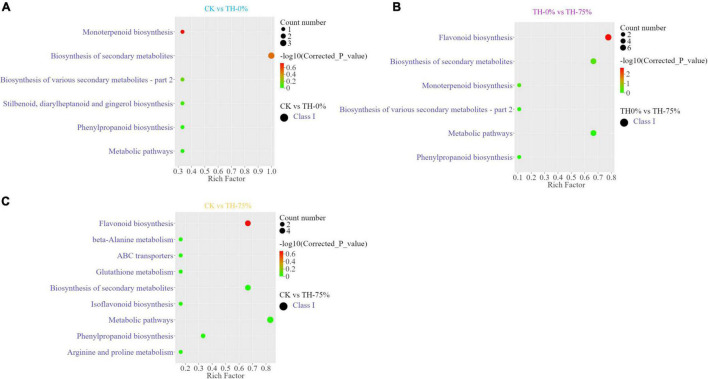
Differentially accumulated metabolites (DAMs) and KEGG enrichment analysis in CK vs TH-0% **(A)**, TH-0% vs TH-75% **(B)**, and CK vs TH-75% **(C)** groups.

Annotated DAMs were mapped to the Kyoto Encyclopedia of Genes and Genomes (KEGG) pathway database^3^, and pathway enrichment analysis was performed. The top enriched KEGG terms of the DAMs detected when CK and TH-0% groups were compared were the monoterpenoid and phenylpropanoid biosynthesis pathways (Figure 5A). The most enriched KEGG terms of the DAMs detected when TH-0% and TH-75% groups were compared were flavonoid and phenylpropane biosynthesis pathways (Figure 5B). The top enriched KEGG terms among the DAMs detected when CK and TH-75% groups were compared were flavonoid, isoflavone, and phenylpropane biosynthesis pathways (Figure 5C). In consequence, DAMs related to monoterpenoid, stilbenoid, diarylheptanoid, gingerol, and phenylpropanoid biosynthesis pathways were mainly enriched under the cold stress treatment. The accumulation of DAMs was enriched in flavonoid, monoterpenoid, and phenylpropane biosynthesis pathways in response to HRW treatment under the cold stress.

### Transcriptome Analysis of HRW-Treated *T. hemsleyanum* Under Cold Stress

**FIGURE 6:**
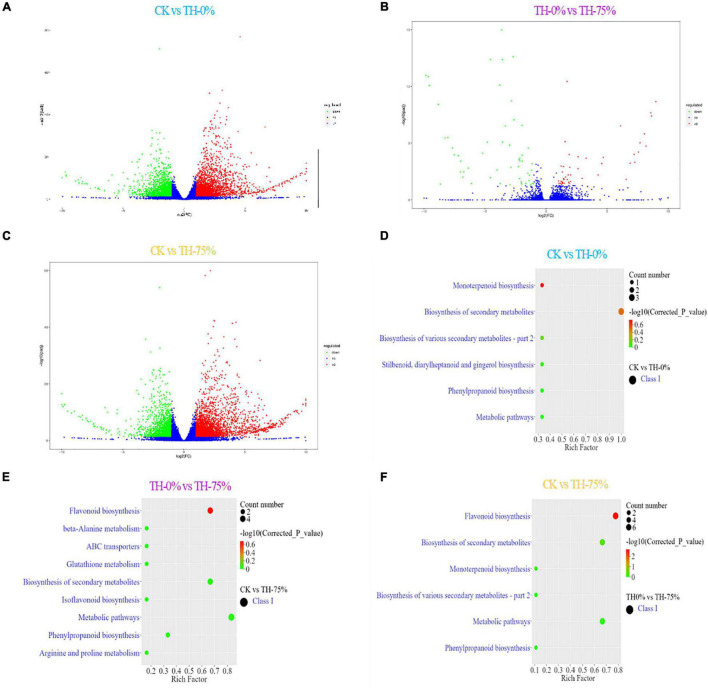
Bar plot showing numbers of DEGs in CK vs TH-0% **(A)**, TH-0% vs TH-75% **(B)**, and CK vs TH-75% **(C)** groups. Bubble plots showing significantly enriched KEGG pathways (*P* < 0.05). Top 10 pathways according to enrichment factor from DEGs in CK vs TH-0% **(D)**, TH-0% vs TH-75% **(E)**, and CK vs TH-75% **(F)** groups. The horizontal axis represents the differential expression multiple, and the vertical axis represents the degree of difference in gene meaning. The red dots indicate the upregulated expressed genes (Log_2_FC ≥ 2), the green dots indicate the downregulated expressed genes (Log_2_FC ≤ 0.5), and the blue dots indicate the non-significantly differentially expressed genes (0.5 < Log_2_FC < 2).

A transcriptomic comparison was carried out to identify differentially expressed genes (DEGs) in CK vs TH-0%, TH-0% vs TH-75%, and CK vs TH-75% comparative groups, respectively. A total of 11,847 DEGs were identified based on transcriptome analysis. A total of 5,869 DEGs were identified in CK vs TH-0% groups, including 3,356 upregulated and 2,513 downregulated genes (Figure 6A). There were 101 DEGs in the TH-0% vs TH-75% groups, including 47 upregulated genes and 54 downregulated genes (Figure 6B). There were 5,877 DEGs in CK vs TH-75% groups, of which 3,738 were upregulated and 2,139 were downregulated (Figure 6C).

The KEGG terms of DEGs between CK and TH-0% groups were mainly enriched in flavonoid, phenylpropanoid, and isoflavonoid biosynthesis pathways (Figure 6D). The significantly enriched KEGG terms of DEGs between TH-0% and TH-75% were monoterpenoid and phenylpropanoid biosynthesis pathways (Figure 6E). The top enriched KEGG terms of the DEGs between CK and TH-75% were flavonoid, phenylpropane, and isoflavone biosynthesis pathways (Figure 6F). To sum up, the above KEGG analysis indicated that DEGs involved in flavonoid, phenylpropane, flavone, flavonol, and isoflavone biosynthesis under cold stress treatment were enriched. DEGs of the phenylpropanoid biosynthesis pathway were particularly enriched in the HRW-treated group.

### Co-expression Network Analysis of DEGs

**FIGURE 7:**
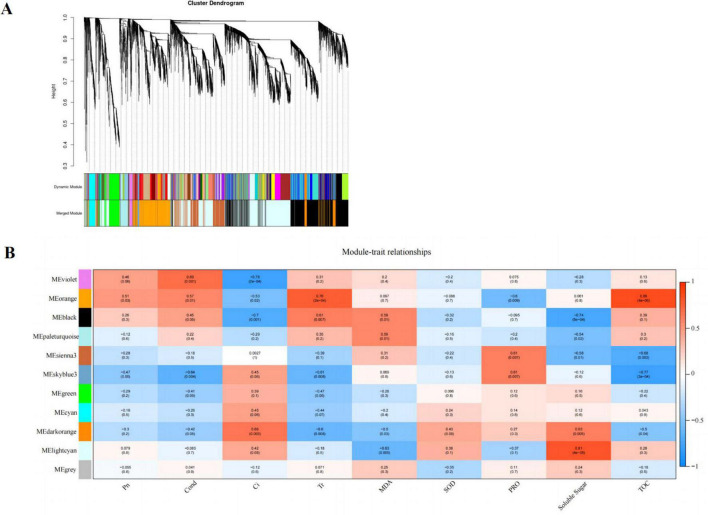
Cluster hierarchy of 24 co-expression modules divided by co-expression network **(A)**; Identification of pathway modules related to photosynthesis and cold tolerance indexes **(B)**. The correlation coefficient between the module and sample is described by the color of each cell at the row-column intersection. Red and blue colors indicate the positive and negative correlations, respectively.

We used weighted gene co-expression network analysis (WGCNA), which can find the modules of highly correlated genes and divide specific genes associated with physiological and biochemical indexes, to identify the key candidate genes involved in physiological and biochemical regulation. WGCNA related these modules to traits, and different expression modules were represented by different colors. The soft threshold of the weighted gene co-regulation network is shown in Figure 7A. In this study, 11,847 DEGs were established into 11 different modules after the clustering and significant analysis (Figure 7B). The MEorange gene module showed a strong correlation with the concentration of T-AOC (*r* = 0.86, *P* < 0.05) and Tr (*r* = 0.76, *P* < 0.05). The MElightcyan gene module showed a strong correlation with the concentration of SS (*r* = 0.81, *P* < 0.05).

Based on the comprehensive analysis of the number of genes and their correlation with cold tolerance indexes, MEorange module was selected as the key gene module for subsequent analysis, which consisted of 828 DEGs. Among the DEGs, a total of 17 genes encoding multiple proteins or enzymes involved in flavonoid synthesis were selected, such as PX, C3′H, CHS (chalcone synthase), IF7MAT (isoflavone 7*-O-*glucoside-6″*-O-*malonyltransferase), and CCR (cinnamoyl-CoA reductase).

### Trend Analysis for All DEGs

**FIGURE 8:**
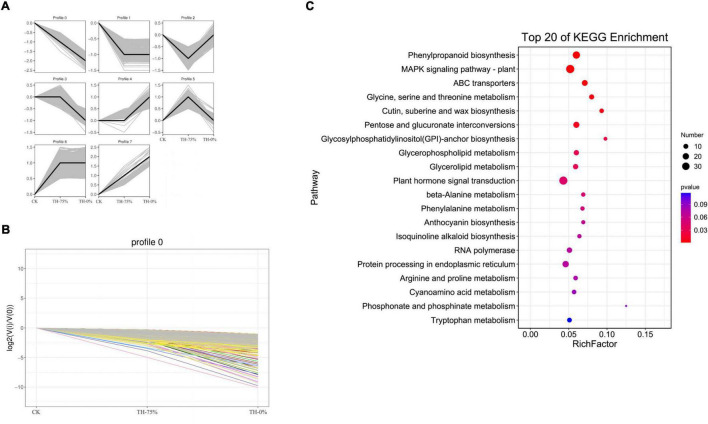
Trend analysis of differentially expressed genes **(A)**, changes in the gene expression level of profile 0 **(B)**, and the pathway of genes in profile 0 can be annotated in the KEGG database **(C)**. From this figure, we can intuitively see the expression changes of genes belonging to this trend, as well as the tightness of aggregation. Each line in the figure represents a gene, and the abscissa is the sample group and the ordinate is the expression change [*log*_2_ (V_i_/V_0_)].

According to the dynamic trend of gene expression in response to HRW treatment under cold stress, DEGs were divided into eight profiles (Figure 8A). Profiles 0,1, 5, and 8 were significantly enriched (*P* < 0.05). Profile 0 was selected because the gene expression level in the CK and TH-75% groups was higher when compared to that in the TH-0% group (Figure 8B). The DEGs in profile 0 were enriched in the phenylpropanoid and anthocyanin biosynthesis pathways (Figure 8C). These results indicated that most of the genes in the phenylpropanoid and anthocyanin biosynthesis pathways decreased under cold stress, but HRW treatment increased the gene expression level of these genes.

**FIGURE 9:**
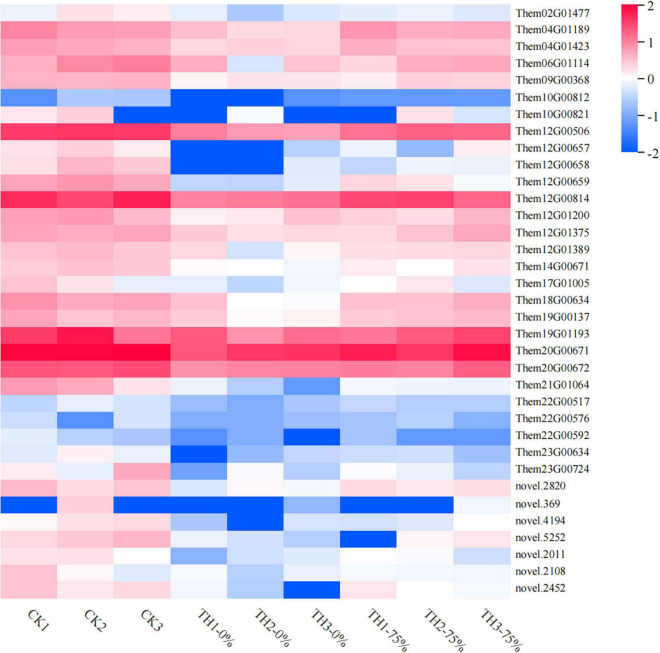
Heat map visualization of the 35 genes in flavonoid metabolism-related pathways selected from profile 0. The color bar represents the log (fold change) values.

Profile 0 contains 1,327 genes, including 35 genes related to the flavonoid synthesis enrichment pathway. A heatmap of 35 DEGs was drawn to represent the effects of cold stress and HRW treatment on *T. hemsleyanum* (Figure 9). The 35 DEGs were identified as those related to phenylpropanoid, anthocyanin, flavonoid, and isoflavonoid biosynthesis pathways, in which the phenylpropanoid biosynthesis pathway was the most annotated. In addition, among the 35 selected DEGs, there were eight genes, including Them12G00506, Them12G00658, Them12G00659, Them12G00814, Them21G01064, novel.2820, novel.4194, and novel.2108, which were members of MEorange module in WGCNA. They were noted to contain only four key enzyme genes in the 8 DEGs, including PX (peroxidase), C3′H [coumaroyl quinate (Coumaroyl shikimate) 3′-monooxygenase], AtBG1 (beta-glucosidase), and 4CL (4-coumarate-CoA ligase). Therefore, it was speculated that the genes of these enzymes played major roles in the transcription and metabolism process of HRW treatment under cold stress.

### Integrated Analysis of Genes and Metabolites Related to Phenylpropanoid, Flavonoid, and Isoflavonoid Biosynthesis in HRW-Treated *T. hemsleyanum* Seedlings Under Cold Stress

**FIGURE 10:**
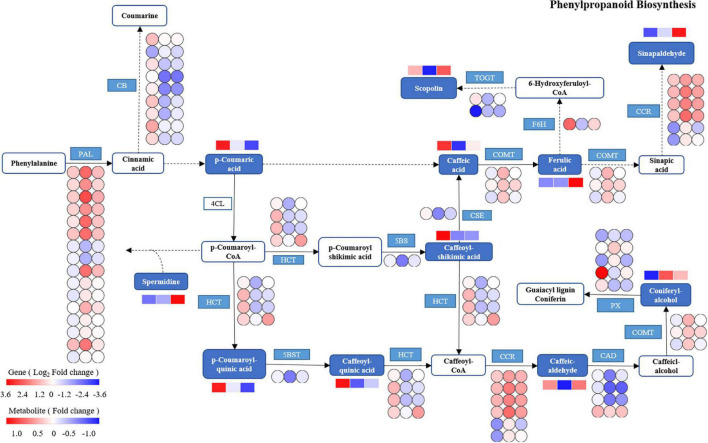
The DEGs and DEMs involved in the phenylpropanoid biosynthesis pathway in response to cold stress. The blue pattern represents the metabolites or genes that changed under CK, TH-0%, and TH-75% groups. The rectangle is divided into three equal parts (the left of the rectangle represents DEGs or DEMs in CK, the middle of the rectangle represents DEGs or DEMs in TH-0%, and the right of the rectangle represents DEGs or DEMs in TH-75%). The color in the rectangle indicates that the genes or metabolites were regulated (red indicates upregulation and blue indicates downregulation). AtBG1: beta-glucosidase; PAL: phenylalanine ammonia-lyase; CCR: cinnamoyl-CoA reductase; PX: peroxidase; HCT: shikimate *O*-hydroxy cinnamoyl transferase; TOGT1: scopoletin glucosyltransferase; F6H: feruloyl-CoA 6-hydroxylase; CSE: caffeoyl shikimate esterase; 5BST: 5*-O-*(4-coumaroyl)-D-quinate 3′-monooxygenase; CAD: cinnamyl-alcohol dehydrogenase; and COMT: caffeic acid 3-*O*-methyltransferase.

Based on the integrated analysis of genes and metabolites, the phenylpropanoid biosynthesis pathway of *T. hemsleyanum* was speculated as shown in Figure 10, and the impacts of HRW treatment on the expression of protease genes and metabolites under cold stress were also displayed. We found that among the 122 DEGs in the phenylpropanoid biosynthesis pathway, 9 AtBG1 and 1 F6H (feruloyl-CoA 6-hydroxylase) were downregulated, while 16 PAL (phenylalanine ammonia-lyase), 6 CCR, and 3 COMT (caffeic acid 3*-O-*methyltransferase) were upregulated in the TH-0% group, compared to the CK group. The expression levels of AtBG1 and F6H of the TH-0% group were lower than in the CK group, while the levels were higher in the TH-75% group when compared to the TH-0% group. On the contrary, the expression levels of PAL, CCR, and COMT of the TH-0% group were higher than in the CK group, while the levels in the TH-75% group were lower than those of the TH-0% group. Compared with CK, the content of downstream metabolite coniferyl alcohol was significantly increased by 75.2% under cold stress, and HRW treatment significantly decreased the enhancement of coniferyl alcohol. The results showed that AtBG1, F6H, PAL, CCR, and COMT were the key genes in the phenylpropanoid biosynthesis pathway in response to cold stress. Their expression changes caused by cold stress could be mitigated by HRW treatment, which also alleviated the synthesis of the key metabolites, especially coniferyl alcohol.

**FIGURE 11:**
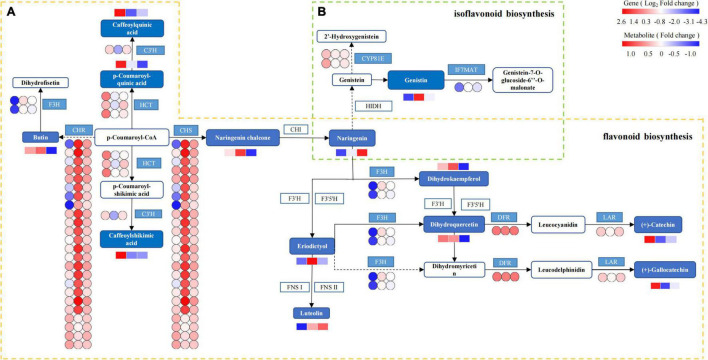
The DEGs and DEMs involved in flavonoid and isoflavonoid biosynthesis in response to cold stress. The flavonoid biosynthesis process is given within the yellow dotted box **(A)**, and the isoflavonoid biosynthesis is within the green dotted box **(B)**. The blue pattern represents the annotated metabolites or genes. The rectangle is divided into three equal parts (the left of the rectangle represents DEGs or DEMs in CK, the middle of the rectangle represents DEGs or DEMs in TH-0%, and the right of the rectangle represents DEGs or DEMs in TH-75%). The color in the rectangle represents that the genes or metabolites are regulated under cold stress (red indicates upregulation and blue indicates downregulation). CHS: chalcone synthase; CHR: chalcone reductase; HCT: shikimate *O*-hydroxycinnamoyl transferase; C3′H: 5-*O*-(4-coumaroyl)-D-quinate 3′-monooxygenase; LAR: leucoanthocyanidin reductase; F3H: naringenin 3-dioxygenase; DFR: bifunctional dihydroflavonol 4-reductase; IF7MAT: isoflavone 7*-O-*glucoside-6″*-O-*malonyltransferase; and CYP81E: isoflavone.

Based on the integrated analysis of genes and metabolites, the flavonoid and isoflavonoid biosynthesis pathway of *T. hemsleyanum* was speculated as shown in Figure 11, and the effects of HRW treatment on protease gene and metabolite expression under cold stress were also shown. The 201 DEGs were found to be related to flavonoid and isoflavonoid biosynthesis. Of them, 24 CHS, 24 CHR (chalcone reductase), and 1 IF7MAT were upregulated in the TH-0% group, compared to the CK group. The results showed that the expression levels of CHS, CHR, and IF7MAT in TH-0% were higher than in CK, and HRW treatment could significantly decrease the enhancement of their expression levels. Compared with CK, the accumulation of eriodictyol in TH-0% was increased significantly by 103.2%, and their content in TH-75% was 41.5% lower than that that observed in TH-0%. The results showed that CHS, CHR, and IF7MAT were the key genes in the flavonoid and isoflavonoid biosynthesis pathways in response to cold stress. Their expression changes caused by cold stress could be mitigated by HRW treatment, which also alleviated the synthesis of key metabolites, especially eriodictyol.

### Real-Time Quantitative PCR Analysis Validation

**FIGURE 12:**
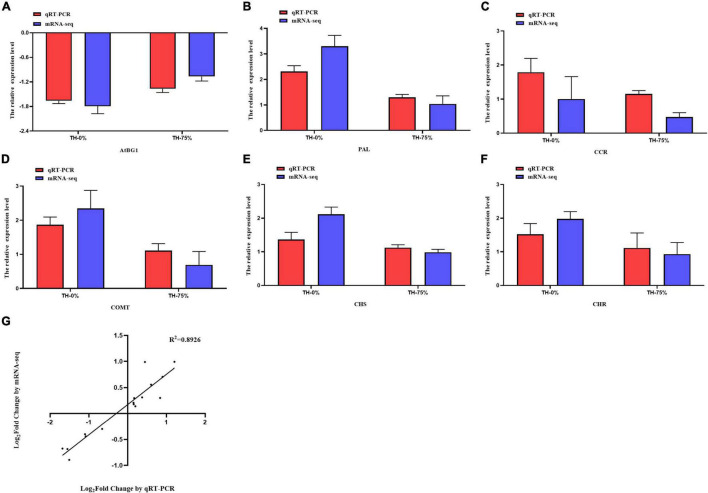
Expression pattern validation **(A–F)** and linear dependence relation between the log_2_ values of the key gene expression ratios obtained from RNA-seq and qRT-PCR **(G).**

To verify the reliability of transcriptome data analysis, we selected eight key genes (ATBG1, PAL, CCR, F6H, COMT, CHS, CHR, and IF7MAT) to detect the transcript levels of the genes by qRT-PCR. This independent evaluation revealed that the patterns of RNA-Seq expressions on these genes were highly consistent with the qRT-PCR data, and a correlation coefficient (R^2^) of 89.26% between the two techniques was obtained (Figure 12).

## Discussion

### HRW Treatment Alleviated the Physiological Responses of *T. hemsleyanum* to Cold Stress

Cold stress has significant effects on a series of important physiological and biochemical processes, such as material metabolism and photosynthetic energy metabolism. Photosynthetic efficiency and antioxidant capacity are the most important parameters in plant adaption to environmental stress, such as Pn, Cond, Ci, Tr, MDA, PRO, SOD, SS, T-AOC, etc. The levels of all the photosynthetic parameters, including Pn, Cond, and Tr, of *Oryza sativa* seedlings were decreased by salinity stress when compared to those observed under normal growth conditions (Sheteiwy et al., 2019). Xie et al. (2022) also reported a significant reduction in Pn, Cond, and Tr values, and a significant increase in Ci level in *T. hemsleyanum* under photovoltaic adaptation. The findings of our study were similar to those reported by these previous studies, that is, Pn, Cond, and Tr were reduced, Ci was provoked, and the photosynthetic sustainability of *T. hemsleyanum* was disrupted when exposed to cold stress conditions. Pn, Cond, and Tr values in the HRW treatment group were significantly higher than in the group of maize seedlings exposed to aluminum stress (Zhao et al., 2017). Chen et al. also found that HRW pretreatment increased Pn and Cond values of cucumber leaves under high-temperature stress (Chen et al., 2017). Our result was consistent with this study, showing that Pn, Cond, and Tr values were increased in HRW treatment under cold stress. Cold stress significantly reduced Pn, which could be a kind of self-protection by plants to reduce stress-induced damage. HRW could enhance the cold tolerance by significantly reducing the stomatal density.

Malondialdehyde is an oxidation end product in plants. Hu et al. found that cold stress enhanced lipid peroxidation in plant cells by increasing MDA accumulation (Hu et al., 2020). Previous studies have proved that the MDA content of *T. hemsleyanum* under cold stress increased with an increase in the treatment time (Peng et al., 2019). In our study, the content of MDA was also increased under cold stress in *T. hemsleyanum*. Pretreatment with HRW decreased the MDA content in cucumber seedlings under salt stress, and HRW inhibited the accumulation of MDA in the rape seedlings exposed to excessive concentrations Ca(NO_3_)_2_, which were consistent with the results obtained in this study (Wei et al., 2021). Moreover, in Wang’s research, compared with the CK treatment, the MDA content was reduced by HRW treatment in Cd-stressed cucumber (Wang et al., 2019). Similarly, in our study, MDA content was significantly decreased by 21.5–41.6% under HRW treatments in *T. hemsleyanum*, compared with the cold stress group. HRW enhanced the active oxygen scavenging capacity, regulated the redox balance in cells, and decreased the content of MDA in plants under stress by reducing the content of active oxygen.

It was considered that the stomatal density of the *Stevia rebaudiana* was enhanced under cold stress (Hajihashemi et al., 2018). Zhang S. S. et al. (2020) reported a significant increase in the stomatal density in the leaf veins of *Dendrobium officinale* in response to cool temperatures. Similarly, our investigation indicated that the stomatal density of *T. hemsleyanum* increased under cold stress. Further results revealed that H_2_ might be involved in the ABA signaling pathway and that HRW promoted stomatal closure for drought tolerance (Xie et al., 2014). Stomatal closure was speculated to be a key step in crop tolerance against osmotic stress and drought (Zhang Y. H. et al., 2020). This study illustrated that HRW might effectively enhance the cold tolerance of *T. hemsleyanum*.

### Secondary Metabolic Regulation Plays an Important Role in Alleviating the Damage of Cold Stress by HRW Treatment

Genes encoding various enzymes can be induced under cold stress, and their signal transduction can reduce the damage caused by stress in plants. They play an extremely important role in plant growth and development. In order to improve the adaptability of plants to cold stress, physiological and biochemical changes occur in plants following HRW treatment. Studies confirmed that cold stress regulation in *Citrus junos* seedlings (Jiang et al., 2021), winter turnip rape (Xu et al., 2018), and rubber tree (Gong et al., 2018) was related to genes mostly involved in phenylalanine, isoflavone, and flavonoid biosynthesis, respectively. In our study, HRW response to cold stress was also mainly related to the enrichment of biosynthetic pathways mentioned above and regulation of related gene families, including PAL, CHS, COMT, AtBG1, and IF7MAT. Under long-term overgrazing and cold stress, PAL expression of *Leymus chinensis* (Wan et al., 2021) and *T. hemsleyanum* (Peng et al., 2019) was significantly upregulated. In our study, cold stress also significantly upregulated the expression of PAL. But the upregulation of PAL was significantly inhibited after HRW treatment, which was consistent with the research results of HRW response to the effect of UV-A irradiation stress on PAL expression of radish sprouts (Zhang et al., 2018). Meanwhile, the expression level of COMT in *T. hemsleyanum* persistently increased after cold stress treatment, which is consistent with the findings of Li et al. on *Ligusticum chuanxiong* (Li et al., 2015). IF7MAT acts downstream of isoflavone biosynthesis and is a key enzyme in this pathway. IF7MAT was significantly upregulated in the roots of *Astragalus membranaceus* exposed to salinity stress (Liu et al., 2022). The changes of some metabolites in the isoflavone biosynthesis pathway may be related to the upregulation of IF7MAT, which is consistent with the expression of this gene in *T. hemsleyanum* under cold stress. CHS, a gene related to eriodictyol synthesis, can be effectively regulated under abiotic stress conditions (cold, darkness, salinity, etc.) (Singh and Kumaria, 2020). For example, CHS expression was significantly upregulated in both *Leymus chinensis* and maize, under long-term overgrazing (Wan et al., 2021) and cold stress conditions (Páldi et al., 2014). In the present study, *T. hemsleyanum* also showed upregulation of CHS expression under cold stress. Lee et al. showed that overexpression of AtBG1 resulted in enhanced drought tolerance of Arabidopsis (Lee et al., 2006), which was consistent with the regulatory role of AtBG1 in the tolerance of *T. hemsleyanum* under cold stress. In conclusion, in our study, cold stress significantly enhanced the expression of the annotated major genes in the biosynthetic pathways of phenylpropanoids, flavonoids, and isoflavonoids exhibiting a general induction effect. We learned that the expression of CHS, PAL, COMT, and IF7MAT genes was significantly upregulated in trichomes under cold stress. Consistently, the enhancement effect was attenuated by HRW treatment, which may effectively reduce oxidative damage by inhibiting the activation of flavonoid-related biosynthetic pathways. In the present study, most of the annotated AtBG1 was downregulated under cold stress. Therefore, it is likely that HRW overexpression of AtBG1 increased ABA levels and cellular resistance, and further studies are needed to verify this speculation.

A previous study showed that secondary metabolites under environmental factor stresses could change the tolerance of plants. Flavanols of *T. hemsleyanum* might play an important role in confronting cold stress. In this study, the cold stress treatment increased the accumulation of flavonoids in *T. hemsleyanum* under cold stress. However, HRW has been reported to restrain secondary metabolism and reduce product accumulation in many plants. For example, HRW treatment slowed down the accumulation of flavonoids and transcript levels of phenylpropanoid pathway genes in fresh-cut Chinese water chestnuts (Li et al., 2022). On the contrary, Gao et al. have assumed that a significant enhancement in the antioxidant capacity and the excessive accumulation of flavonoids played central roles in the tolerance of Medicago sativa seedlings to UV-B (Gao et al., 2019). HRW treatment was reported to significantly enhance flavonoid biosynthesis of alfalfa under UV-B stress (Hong et al., 2014). But in our study, HRW treatment inhibited the accumulation of coniferyl alcohol and eriodictyol, which may be due to the redirection of metabolic fluxes in branches of the phenylpropane pathway in response to cold stress. Zhuang et al. (2021) indicated that the coniferyl alcohol content of *H. vernicosa* increased, which indicated that the antioxidant system of *H. vernicosa* was activated and coniferyl alcohol was synthesized to improve its cold tolerance. This result is contrary to our findings, so we speculate that differences in plant species and abiotic stress types may be responsible for this contradiction. HRW treatment effectively reduced oxidative damage and inhibited the activation of the phenylpropanoid pathway, thus slowing down the accumulation of coniferyl alcohol and eriodictyol.

The analysis confirmed that the effect of cold stress on the activity of phenylpropanoid biosynthesis pathway-related enzyme (AtBG1) was reversed by HRW, leading to a decrease in the content of coniferyl alcohol related to phenylalanine synthesis. The positive effect of cold stress on flavonoid biosynthesis pathway-related enzyme (PAL, CHS, and COMT) activity was reversed by HRW, resulting in a decrease in the accumulation of eriodictyol. HRW treatment reversed the positive effect of the isoflavonoid biosynthetic pathway-related enzyme IF7MAT.

## Data Availability Statement

The datasets presented in this study can be found in online repositories. The names of the repository/repositories and accession number(s) can be found below: National Center for Biotechnology Information (NCBI) BioProject database under accession number: PRJNA816565.

## Author Contributions

YL wrote the manuscript and designed the experiments. JP performed the physiological indicators determination. SN performed the KEGG Pathways enrichment analysis. BX performed the RNA extraction and quality determination and carried out the analysis. KC and XP conceived and coordinated the project. All authors have read and approved the manuscript.

## Conflict of Interest

The authors declare that the research was conducted in the absence of any commercial or financial relationships that could be construed as a potential conflict of interest.

## Publisher’s Note

All claims expressed in this article are solely those of the authors and do not necessarily represent those of their affiliated organizations, or those of the publisher, the editors and the reviewers. Any product that may be evaluated in this article, or claim that may be made by its manufacturer, is not guaranteed or endorsed by the publisher.

## Footnotes

1
http://www.anpel.com.cn/

2
http://www.ncbi.nlm.nih.gov/geo/

3
http://www.kegg.jp/kegg/pathway.html

## Supplementary Material

Supplementary Table 1 All differentially accumulated metabolites in CK, TH-0%, and TH-75% groups.

Supplementary Table 2 All differentially expressed genes in CK, TH-0%, and TH-75% groups.
