# Supplementary figures and images for: Transcriptome and Metabonomics Combined Analysis Revealed the Defense Mechanism Involved in Hydrogen-Rich Water-Regulated Cold Stress Response of Tetrastigma hemsleyanum
Source: Front Plant Sci. 2022 Jun 23;13:889726. doi: 10.3389/fpls.2022.889726 (PMC9260428; doi:10.3389/fpls.2022.889726)

**A**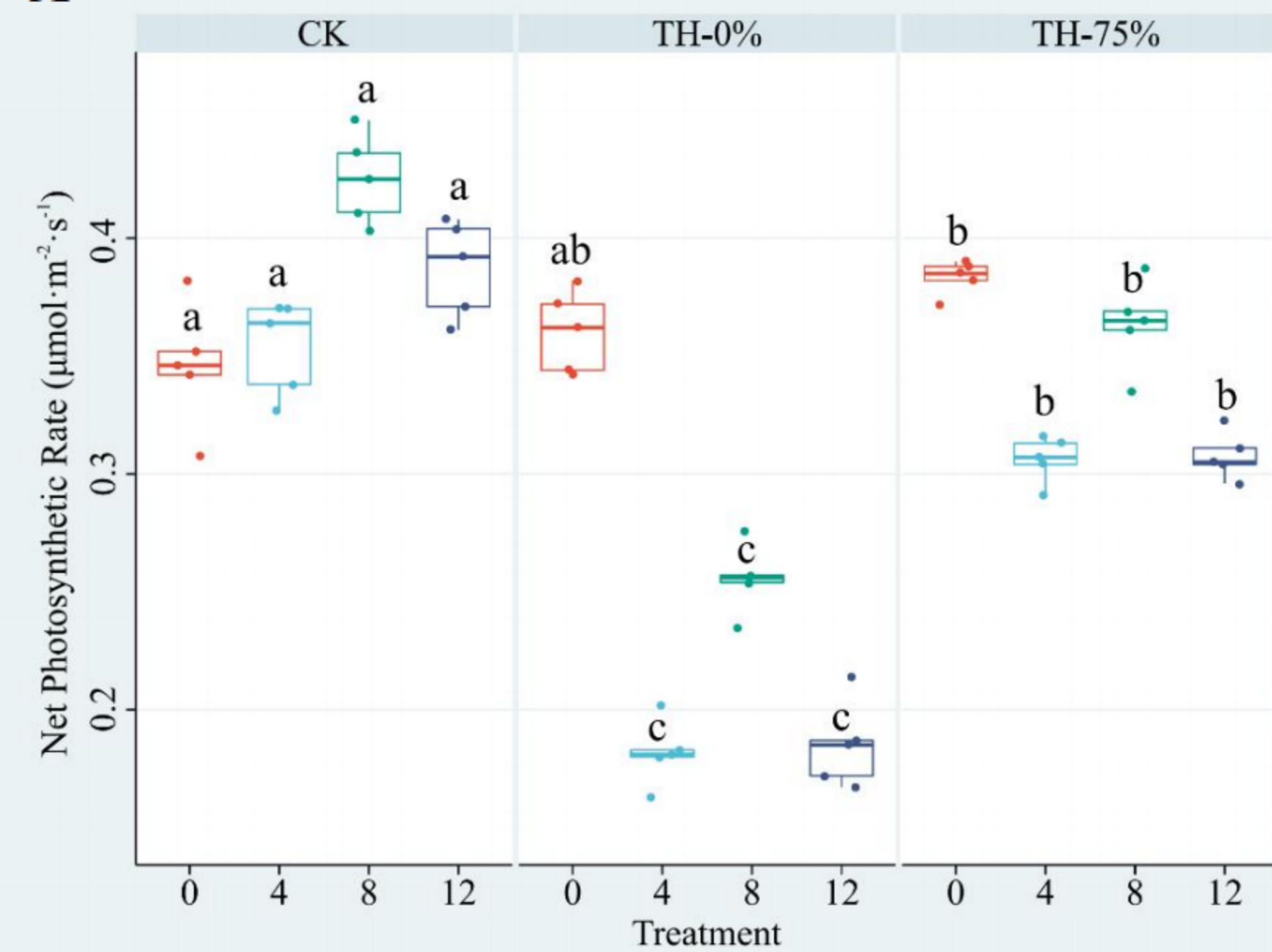**B**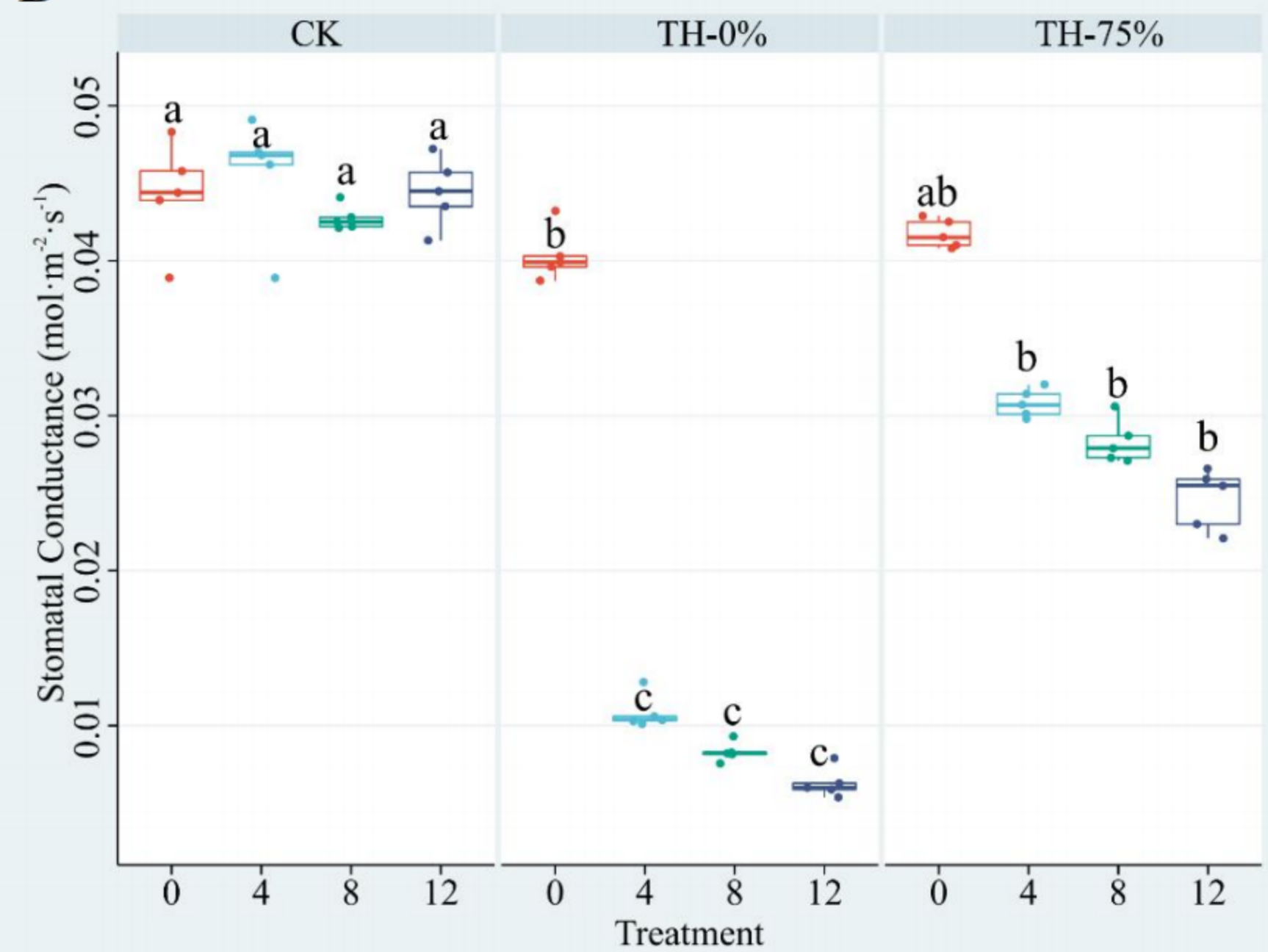**C**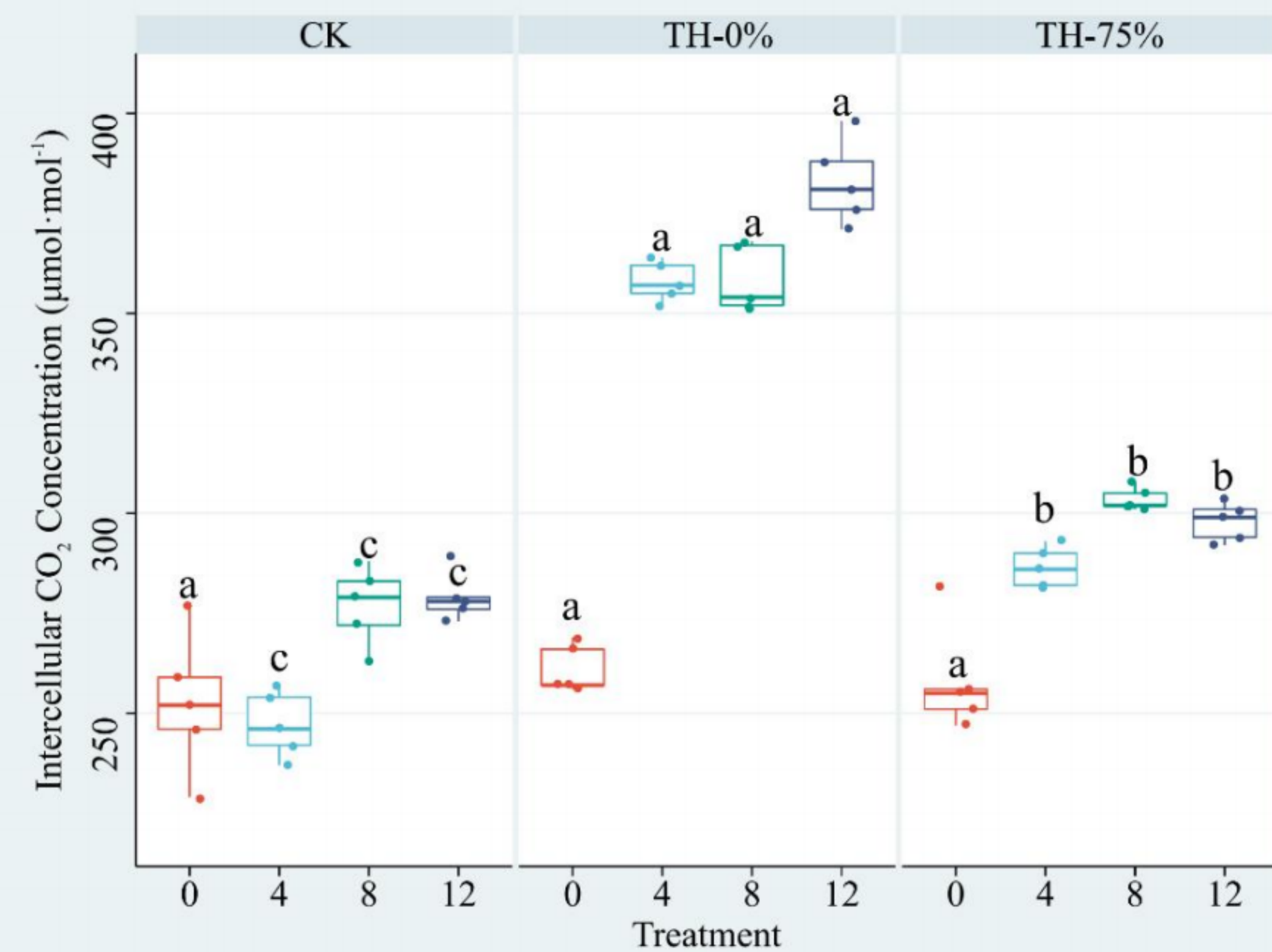**D**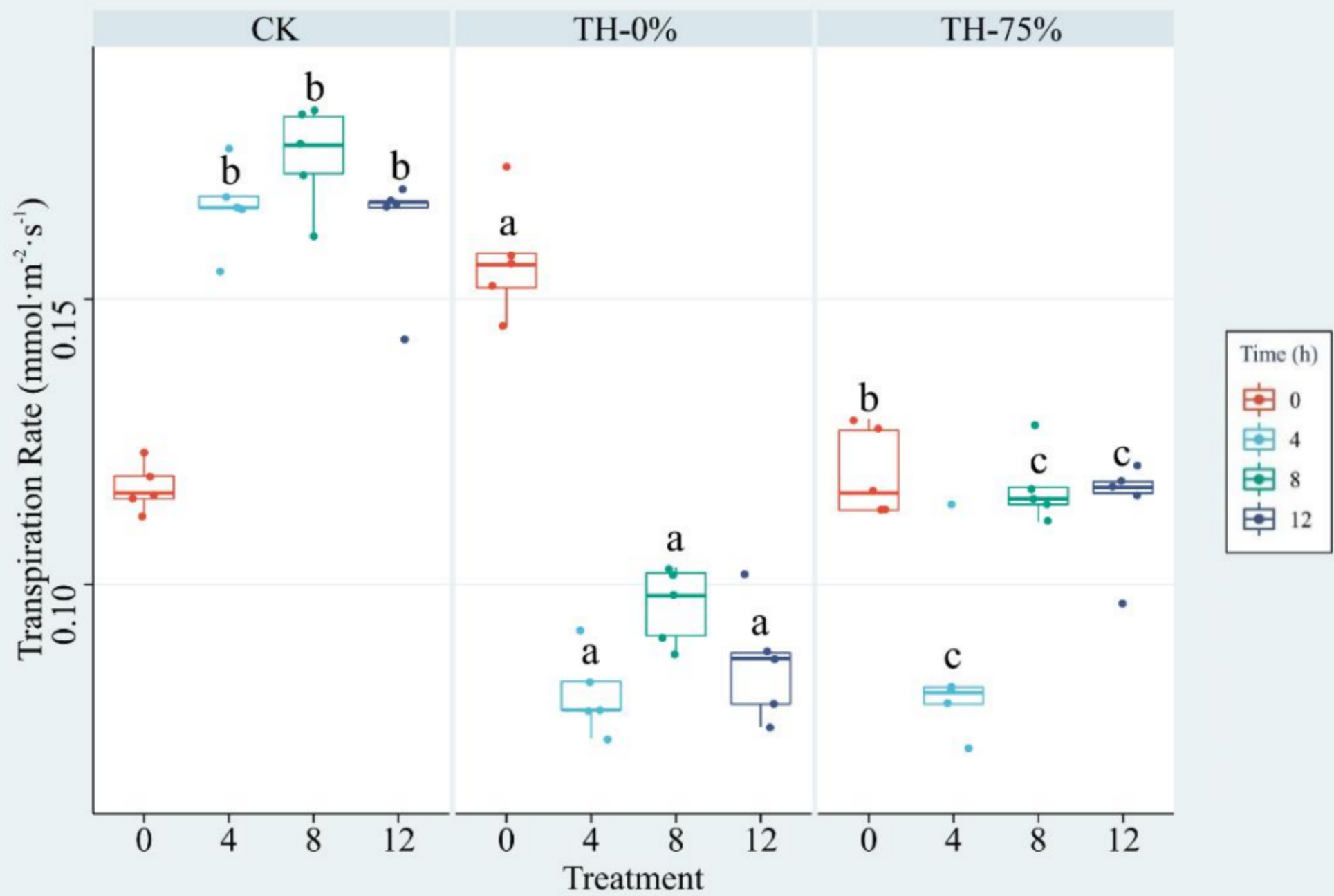

Supplement: Supplementary file 1 [file Image_1.PDF]

**A**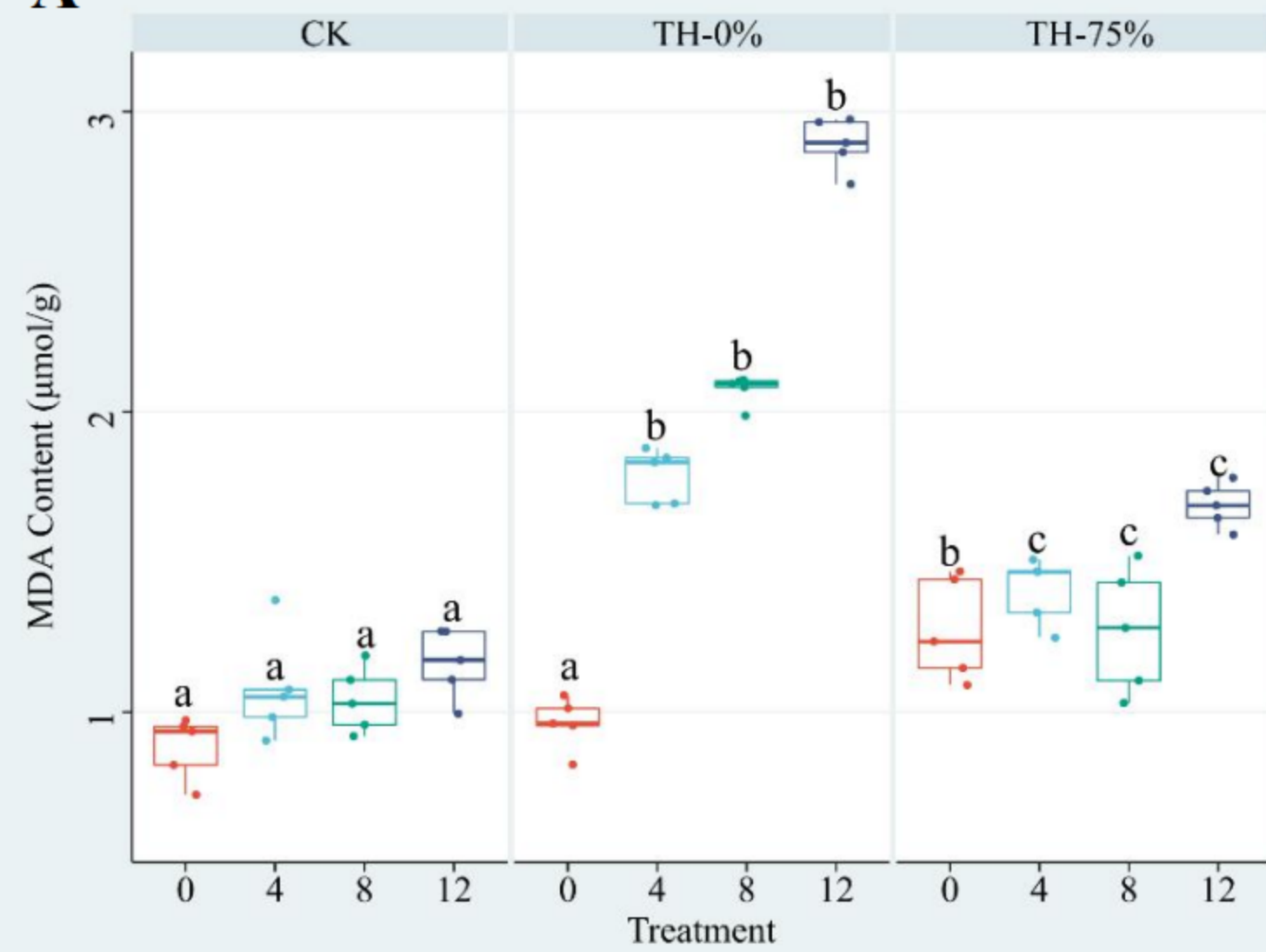**B**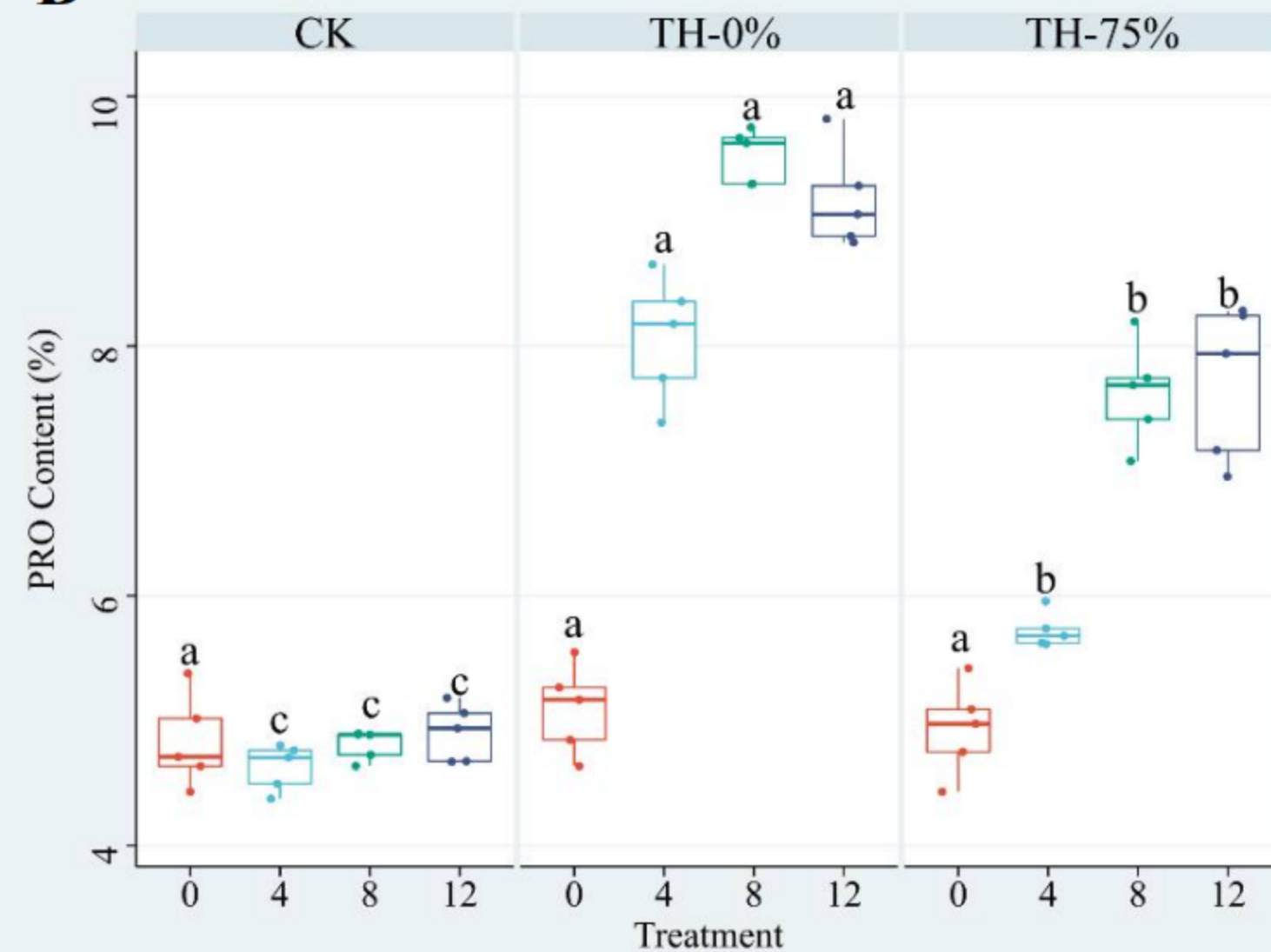**E**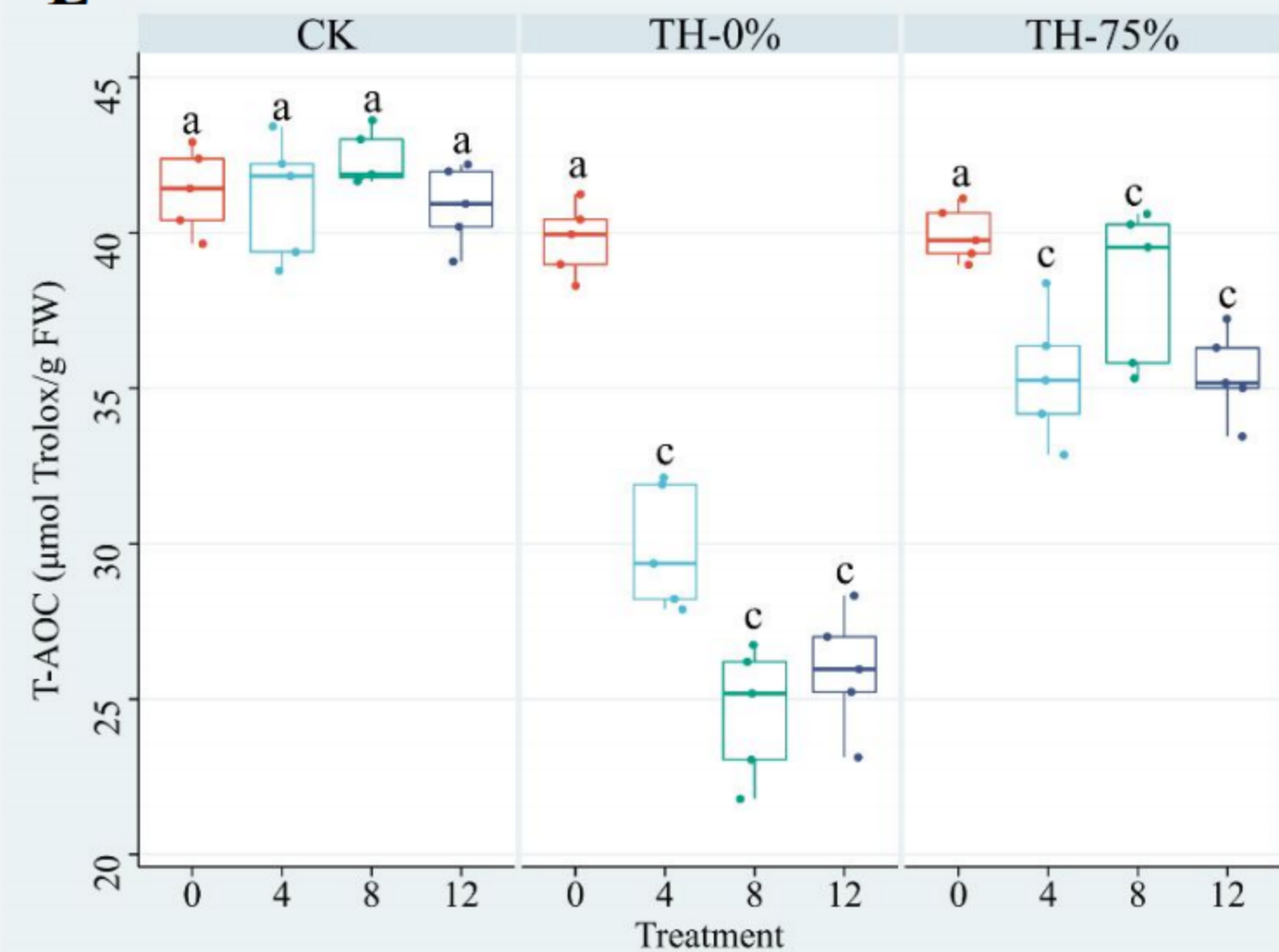**C**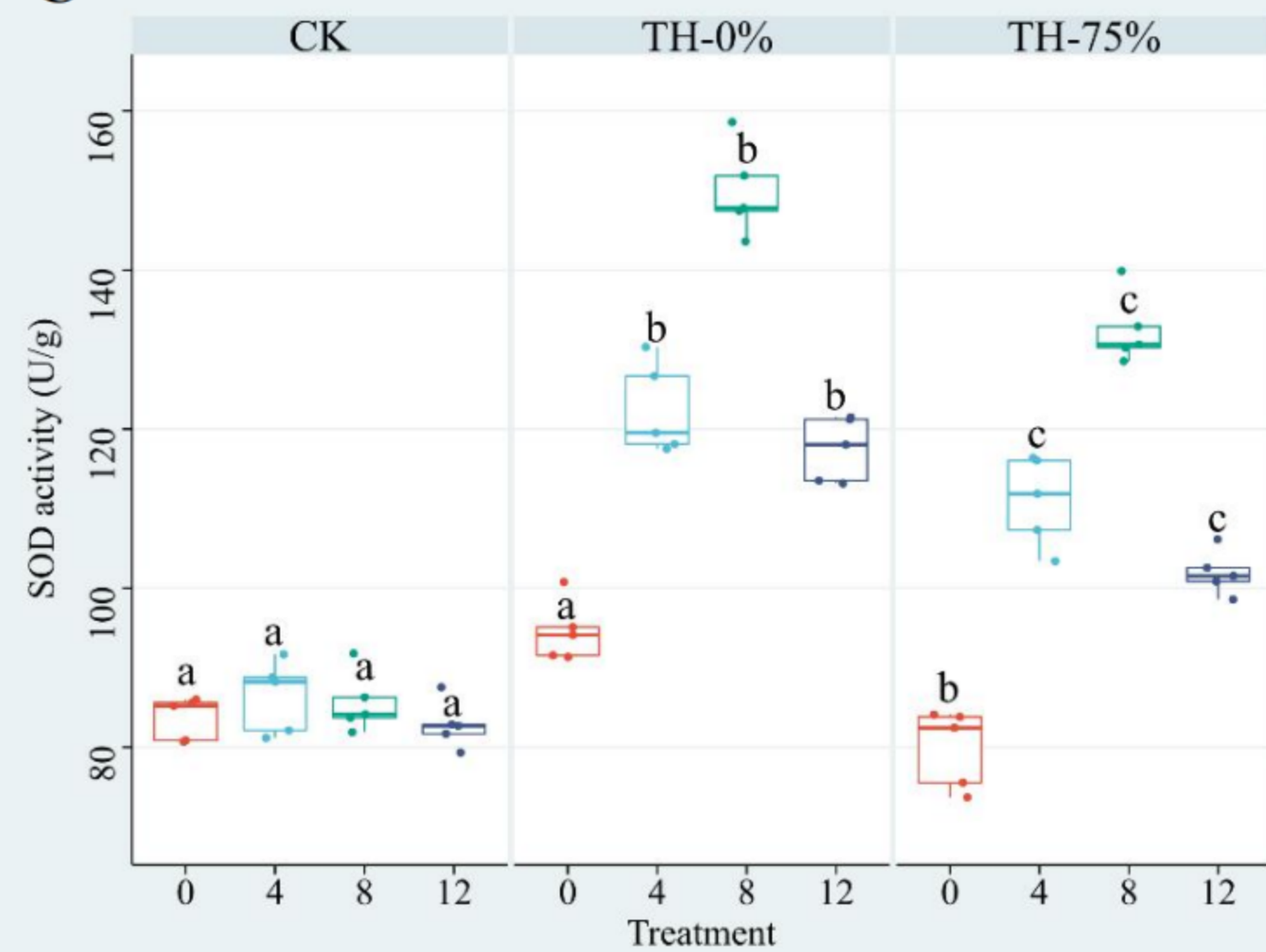**D**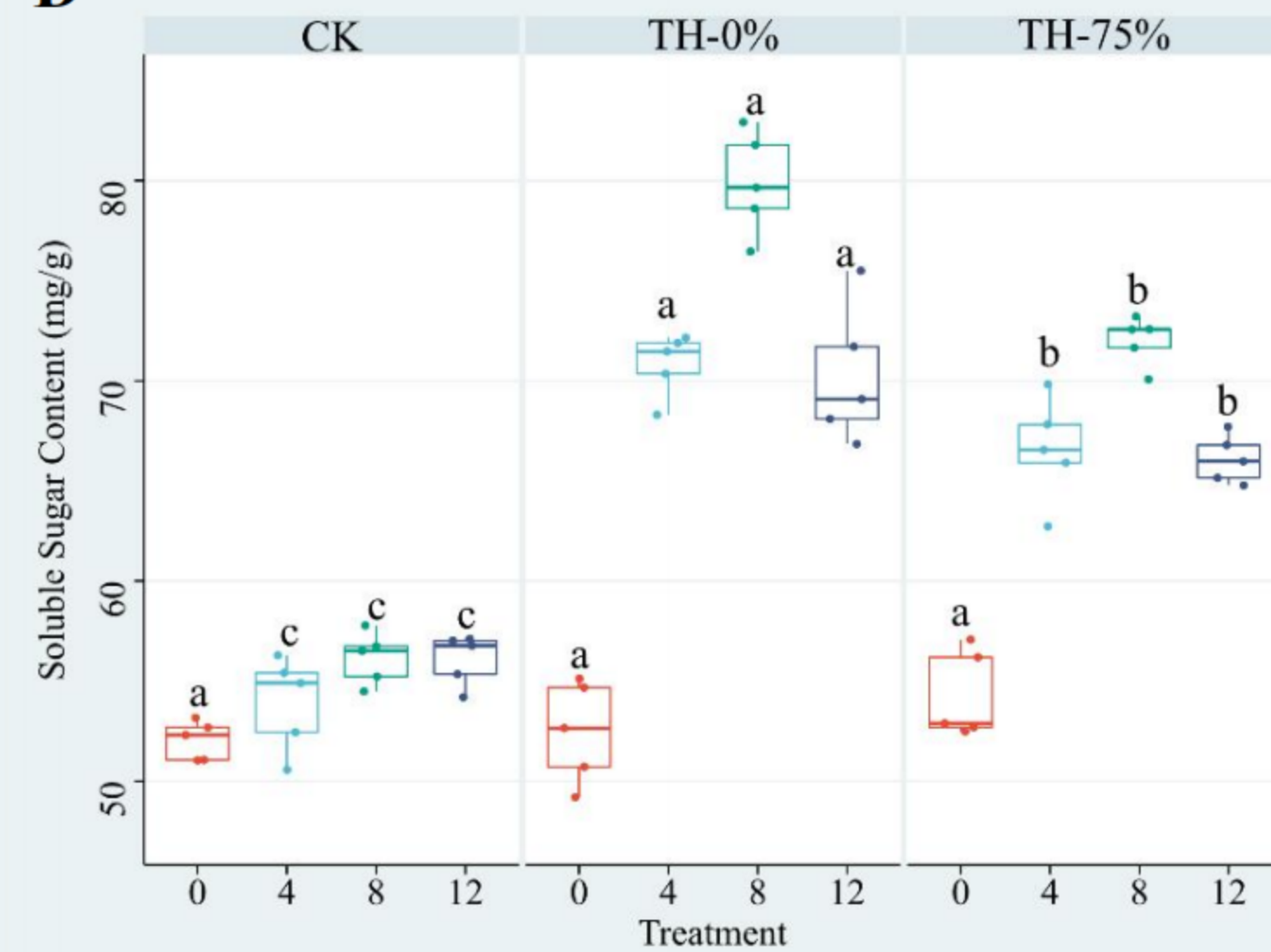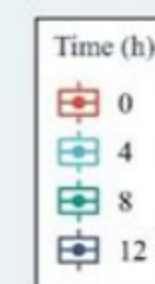

Supplement: Supplementary file 2 [file Image_2.PDF]

**A**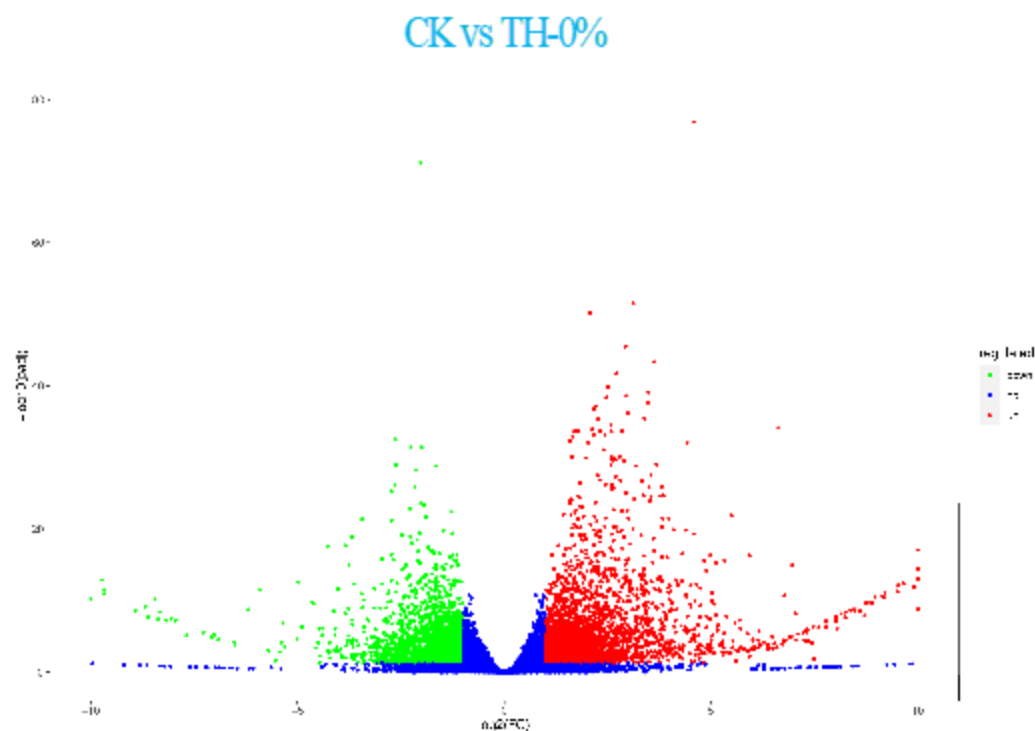**B**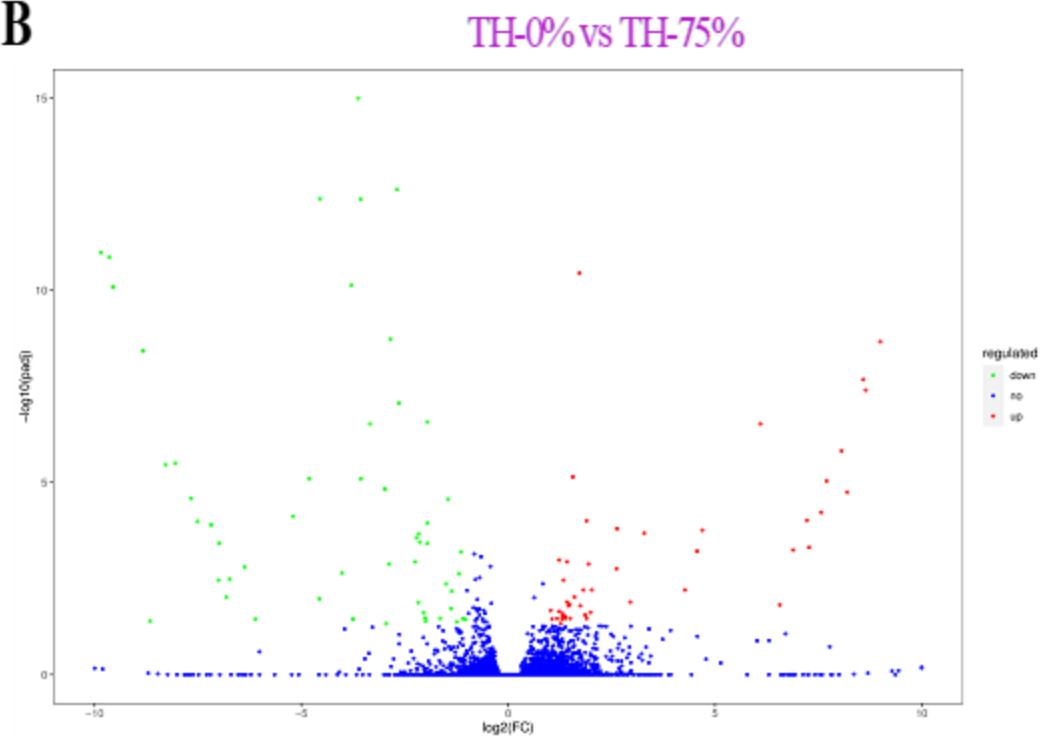**C**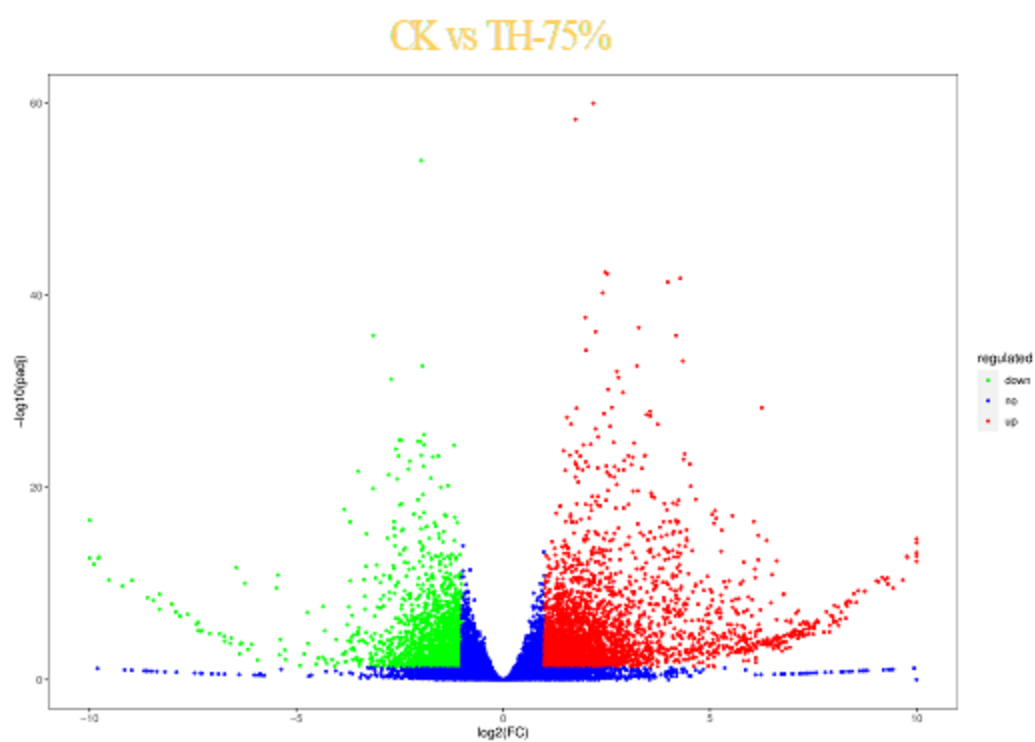**D**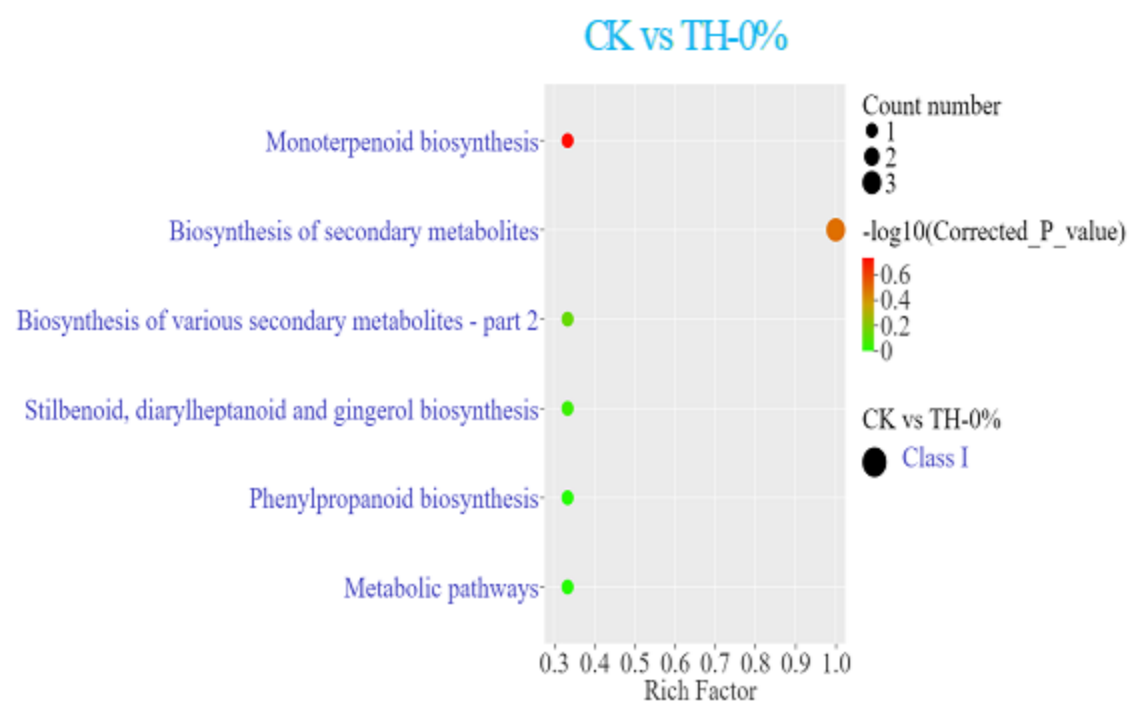**E**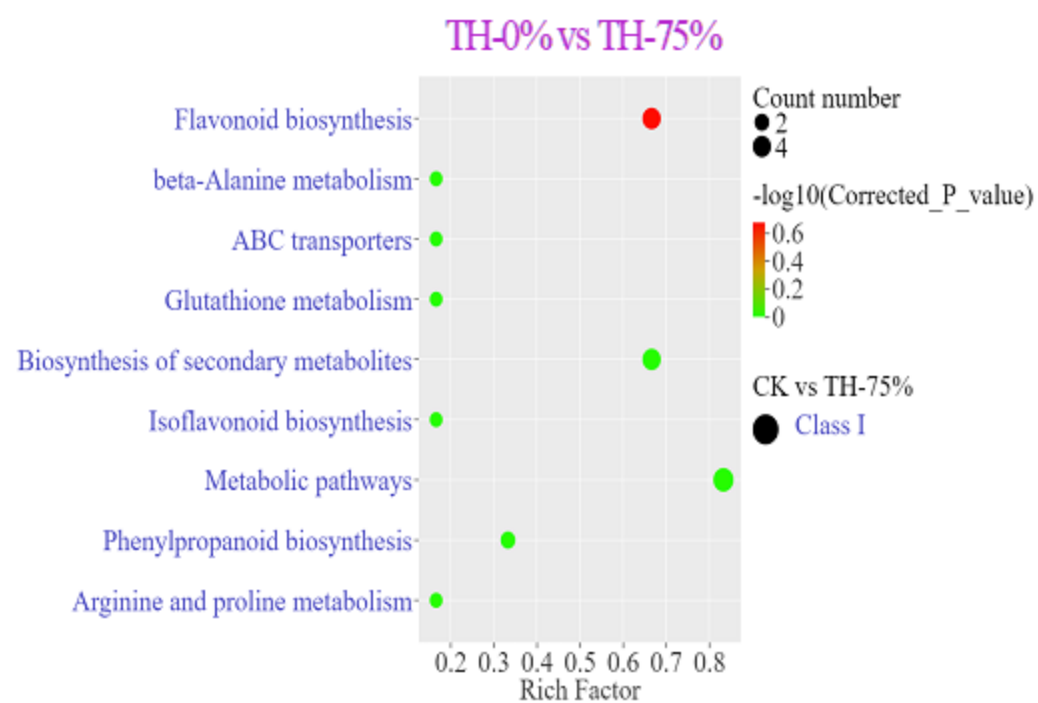**F**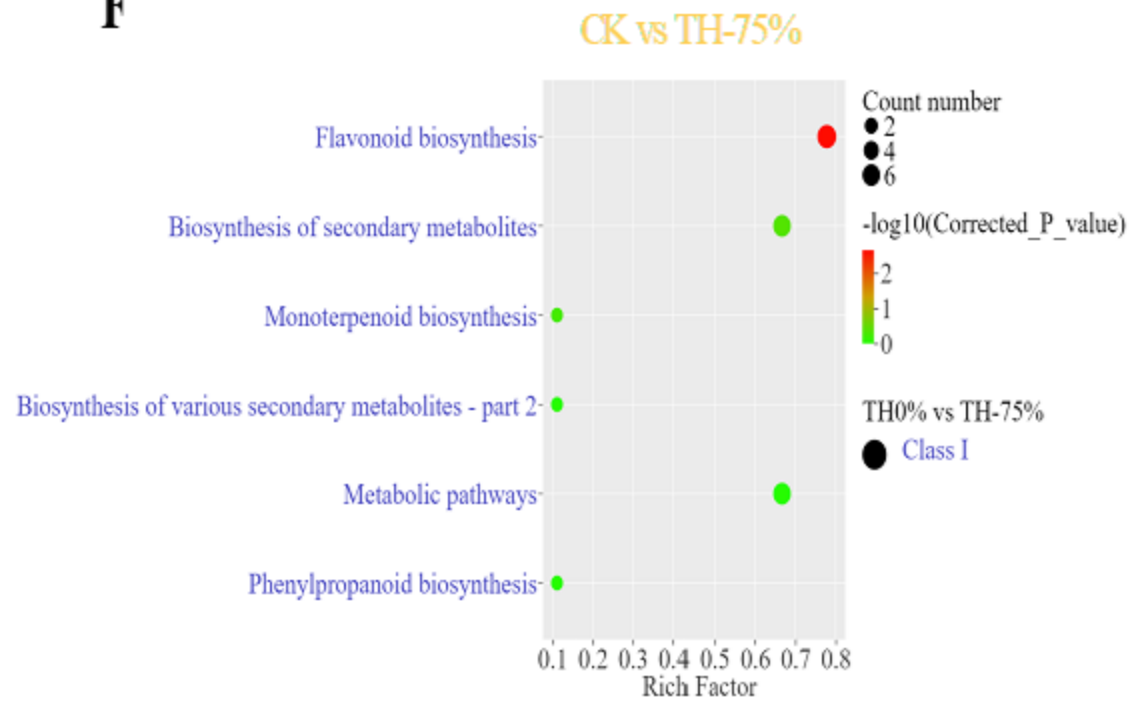

Supplement: Supplementary file 3 [file Image_3.PDF]
